# Comparative genetic mapping and a consensus interspecific genetic map reveal strong synteny and collinearity within the *Citrus* genus

**DOI:** 10.3389/fpls.2024.1475965

**Published:** 2024-12-16

**Authors:** Patrick Ollitrault, Barbara Hufnagel, Franck Curk, Aude Perdereau, Pierre Mournet, Maëva Miranda, Gilles Costantino, Yann Froelicher, Mônica Alves, Maria Angeles Forner Giner, Malcolm W. Smith, Pablo Aleza, François Luro, Nelson A. Wulff, Leandro Peña, Raphaël Morillon, Arnaud Lemainque

**Affiliations:** ^1^ Unité Mixte de Recherche Amélioration Génétique et Adaptation des Plantes méditerranéennes et tropicales (UMR AGAP), Institut Agro, Centre de coopération Internationale en Recherche Agronomique pour le Développement (CIRAD), Institut National de Recherche pour l’Agriculture, l’Alimentation et l’Environnement (INRAE), University of Montpellier, Montpellier, France; ^2^ Unité Mixte de Recherche Amélioration Génétique et Adaptation des Plantes méditerranéennes et tropicales (UMR AGAP), Centre de coopération Internationale en Recherche Agronomique pour le Développement (CIRAD), Montpellier, France; ^3^ Unité Mixte de Recherche Amélioration Génétique et Adaptation des Plantes méditerranéennes et tropicales (UMR AGAP), Centre de coopération Internationale en Recherche Agronomique pour le Développement (CIRAD), Petit-Bourg, France; ^4^ Unité Mixte de Recherche Amélioration Génétique et Adaptation des Plantes méditerranéennes et tropicales (UMR AGAP), Institut National de Recherche pour l’Agriculture, l’Alimentation et l’Environnement (INRAE), Institut Agro, Centre de coopération Internationale en Recherche Agronomique pour le Développement (CIRAD), University of Montpellier, Montpellier, France; ^5^ Genoscope, Institut de Biologie François-Jacob, Commissariat à l’Energie Atomique (CEA), Université Paris-Saclay, Evry, France; ^6^ Unité Mixte de Recherche Amélioration Génétique et Adaptation des Plantes méditerranéennes et tropicales (UMR AGAP), Institut National de Recherche pour l’Agriculture, l’Alimentation et l’Environnement (INRAE), Institut Agro, Centre de coopération Internationale en Recherche Agronomique pour le Développement (CIRAD), University of Montpellier, San Giuliano, France; ^7^ Unité Mixte de Recherche Amélioration Génétique et Adaptation des Plantes méditerranéennes et tropicales (UMR AGAP), Centre de coopération Internationale en Recherche Agronomique pour le Développement (CIRAD), San Giuliano, France; ^8^ Fundo de Defesa da Citricultura, Araraquara, Brazil; ^9^ Departamento de Citricultura y Producción Vegetal, Instituto Valenciano de Investigaciones Agrarias (IVIA), Moncada, Valencia, Spain; ^10^ Department of Agriculture and Fisheries, Bundaberg Research Station, Bundaberg, QLD, Australia; ^11^ Instituto de Biologia Molecular y Celular de Plantas – Consejo Superior de Investigaciones Científicas, Universidad Politécnica de Valencia, Valencia, Spain

**Keywords:** citrus, genetic map comparison, genotyping by sequencing, comparative genomic analysis, recombination landscape, skewed segregation

## Abstract

**Introduction:**

Useful germplasm for citrus breeding includes all sexually compatible species of the former genera *Citrus, Clymenia, Eremocitrus, Fortunella, Microcitrus, Oxanthera*, and *Poncirus*, now merged in the single *Citrus* genus. An improved knowledge on the synteny/collinearity between the genome of these different species, and on their recombination landscapes, is essential to optimize interspecific breeding schemes.

**Method:**

We have performed a large comparative genetic mapping study including several main clades of the *Citrus* genus. It concerns five species (*C. maxima, C. medica, C. reticulata, C. trifoliata* and *C. glauca*), two horticultural groups resulting from interspecific admixture (clementine and lemon) and two recent interspecific hybrids (*C. australis x C. australasica and C. maxima x C. reticulata*). The nine individual genetic maps were established from GBS data of 1,216 hybrids.

**Results and discussion:**

The number of SNPs mapped for each parent varies from 760 for *C. medica* to 4,436 for the *C. maxima x C. reticulata* hybrid, with an average of 2,162.3 markers by map. Their comparison with *C. clementina* v1.0 assembly and inter-map comparisons revealed a high synteny and collinearity between the nine genetic maps. Non-Mendelian segregation was frequent and specific for each parental combination. The recombination landscape was similar for the nine mapped parents, and large genomic regions with very low recombination were identified. A consensus genetic map was successfully established. It encompasses 10,756 loci, including 7,915 gene-based markers and 2,841 non-genic SNPs. The anchoring of the consensus map on 15 published citrus chromosome-scale genome assemblies revealed a high synteny and collinearity for the most recent assemblies, whereas discrepancies were observed for some older ones. Large structural variations do not seem to have played a major role in the differentiation of the main species of the Citrus genus. The consensus genetic map is a useful tool to check the accuracy of genome assemblies, identify large structural variation and focus on analyzing potential relationships with phenotypic variations. It should also be a reference framework to integrate the positions of QTLs and useful genes identified in different analyses.

## 1 Introduction

**Figure 1:**
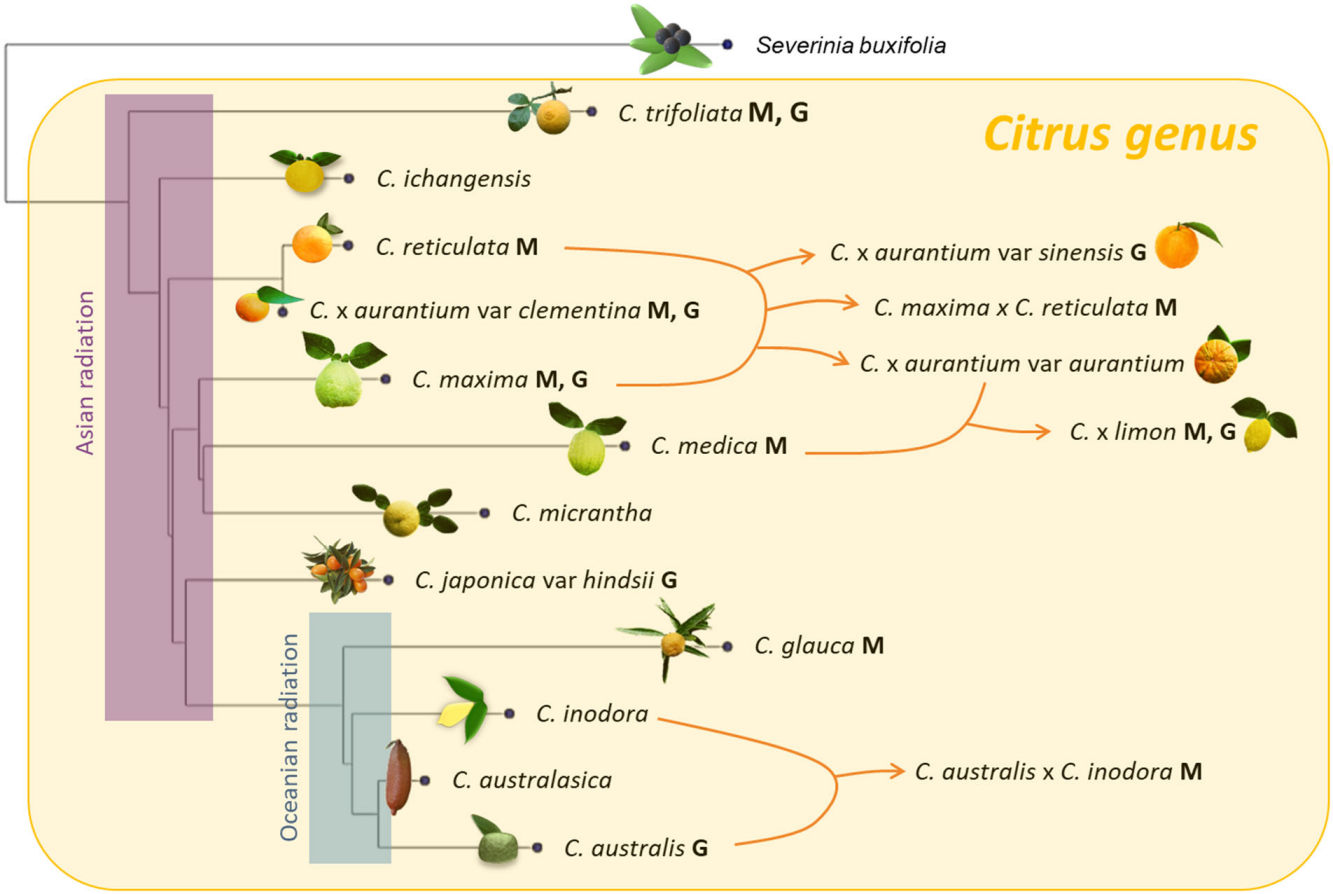
Phylogenetic organization of the Citrus genus and location of the accessions considered in this paper for genetic mapping (M) and genome assemblies (G). The phylogenetic tree was constructed by Minimum Evolution (ME) approach (Desper and Gascuel, 2002) and is based on 181207 diallelic SNPs identified from variant calling of WGS data on the *C. clementina* V1.0 reference genome after filtration for a minimum distance of 1kb between successive SNPs (our unpublished data). The Asian radiation was estimated by Wu et al. (2018) to have occurred in the late Miocene (6–8 Ma) and Oceanian radiation during the early Pliocene epoch (around 4 Ma).

Cultivated citrus and related sexually compatible species constitute a highly polymorphic group whose taxonomic treatment is still controversial. This group is part of the Citrinae subtribe in the Citreae tribe of the aurantioideae subfamily. Swingle and Reece (1967) proposed to subdivide the Citrinae into three groups. According to their taxonomic system that is still widely used, one group, the “true citrus,” includes the *Citrus* genus with most cultivated species and five other genera: *Poncirus*, *Fortunella*, *Eremocitrus*, *Microcitrus* and *Clymenia*. Within *Citrus*, five ancestral species of the actual horticultural groups are clearly identified by phylogenetics (Curk et al., 2016) and phylogenomic studies (Wu et al., 2018; Ahmed et al., 2019): *C. maxima* (pummelos), *C. medica* (citrons), *C. reticulata* (mandarins) and two wild species classified in the subgenus papeda by Swingle and Reece: *C. micrantha* and *C. ichangensis*. The other horticultural group with high economic importance such as sweet-and-sour oranges, grapefruits, lemons and limes results from admixture between these ancestral taxa. However, there is biological evidence that is inconsistent with the circumscription of the genus *Citrus*, as proposed by Swingle and Reece (1967). The different species of the other true citrus genera display sexual compatibility with the *Citrus* species (Iwamasa and Nito, 1988). Moreover, chloroplast and nuclear phylogenetic studies (Bayer et al., 2009; Carbonell-Caballero et al., 2015; Wu et al., 2018) reveal that all “true citrus” species plus *Oxanthera* species constitute a monophyletic clade whose internal organization does not fit with the Swingle and Reece classification. Wu et al. (2018) proposed a first radiation of the true citrus in the late Miocene (6–8 Ma) and a more recent radiation between Australian species occurring during the early Pliocene epoch, around 4 Ma (**Figure 1**). The sexual compatibility and the phylogenetic studies support the proposal of Mabberley (1998) and Zhang and Mabberley (2008) to integrate *Poncirus*, *Fortunella*, *Microcitrus*, *Eremocitrus* and *Clymenia* into the genus *Citrus*. According to chloroplastic phylogeny, the *Oxanthera* species should also be integrated into the *Citrus* genus to respect its monophyletic status (Ollitrault et al., 2020; Mabberley, 2022). In this paper, we adopted the Zhang and Mabberley concept of the *Citrus* genus for the former species of the Swingle and Reece true citrus group (Swingle and Reece, 1967). For horticultural groups resulting from interspecific admixture, we retained the trinomial taxonomic system proposed by Ollitrault et al. (2020) that provides an unambiguous conceptual framework for *Citrus* classification based on the phylogenomic information. All considered *Citrus* species are diploid containing 2n=18 chromosomes.

Sources for adaptation to abiotic stresses and tolerance/resistance to pest and diseases are dispersed in the different subclades of the *Citrus* genus. According to the review of Krueger and Navarro (2007), tolerance to salinity is present in Rangpur lime (*C.* x *limon* var. *limonia* Osb.), Cleopatra mandarin (*C. reticulata* var. *reshni*) and Alemow (*C.* x *aurantiifolia* var. *macrophylla* Wester). *C. glauca* and, at lower level, the Rangpur lime displays tolerance to water deficit. Adaptation to iron chlorosis is found in Rough lemon (*C.* x *limonia* var. *jambhiri* Lush), Volkamer lemon (*C.* x *limonia* var. *volkameriana* Ined) and Nasnaran (*C.* x *amblycarpa* (Hassk.) Ochse and Alemow. The Satsuma mandarins (*C.* × *aurantium* var. *unshiu* ined.), the kumquats (*C. japonica* (Thunb.) Swing.) and trifoliate orange (*C. trifoliata* (L.) Raf.) are cold tolerant. Sour orange (*C. x aurantium* var. *aurantium*), Volkamer lemon, Nasnaran, trifoliate orange and certain pummelos and mandarins are tolerant to *Phytophthora* sp. Tolerance to the African citrus cercosporiosis (fungal disease due to *Phaemularia angolensis* De Carvalho and O. Mendes P.M. Kirk) is found in pummelos, lemons (*C. x limon* var. *limon*) and Satsuma and Beauty (*C.* × *aurantium* var. *tangerina* ined.) mandarins. The susceptibility to another fungal disease, *Alternaria alternata* of tangerine, is only found in a limited set of mandarins (Cuenca et al., 2016) related to the Dancy variety (*C.* × *aurantium* var. *tangerina* ined.). Behavior against citrus tristeza virus (CTV) is highly variable. Trifoliate orange was considered for a long time as immune but some resistance-breaking or “RB” strains of CTV have been described by Harper et al. (2010) to cause systemic infection. Partial resistance to CTV is found in some pummelos and kumquats and tolerance in different germplasm used as rootstocks such as Nasnaran, Cleopatra mandarin, Rangpur lime, Rough lemon and Volkamer lemon. Different levels of tolerance to citrus canker (bacteriosis due to *Xanthomonas citri* subsp*. citri*) are found in kumquats, Satsuma and Dancy mandarins. Trifoliate orange is a source of resistance to the nematode *Tylenchulus semipenetrans* Cobb and has been described in some studies as tolerant to Huanglongbing [the most devastating citrus disease due to the bacteria *Candidatus Liberibacter* sps.; (Folimonova et al., 2009; Albrecht and Bowman, 2011; Ramadugu et al., 2016)] and its vector (George and Lapointe, 2019). However, the determinism and consistence of the resistance/tolerance of trifoliate orange to HLB are still debated today (Alves et al., 2021). Recently, complete resistances to Huanglongbing were described in *C. australis, C. glauca, C. warburgiana, C. wintersii*, and some of their hybrids (Alves et al., 2021). Therefore, under its new definition, the entire *Citrus* genus can be considered the fundamental germplasm to improve citrus by sexual hybridization, and all species need to be integrated into international efforts to map and exploit the citrus genome.

Meiotic recombination is a major component of the evolution of sexually reproducing species, and the resulting allele shuffling between the parental homolog chromosomes is fundamental in sexual breeding strategies. The crossover distribution is heterogeneous within and among chromosomes (Boulton et al., 1997; Myers et al., 2010), but also between species (Dumont and Payseur, 2011) and genotypes (Kong et al., 2010; Kawakami et al., 2017), and defines the recombination landscape. The centromeric and pericentromeric genome regions generally present a very low recombination level, where allele shuffling is strongly hampered in comparison with other genomic regions. High inter-homolog sequence divergence or structural variation in interspecific hybrids can also strongly affect the recombination rates in non-centromeric regions. From an evolutionary point of view, sexual recombination creates new haplotypes, which in turn can have an impact on natural selection (Hill and Robertson, 2007; Maynard and Haigh, 2007) and, consequently, on the distribution of diversity on a genome-wide scale (Ellegren and Galtier, 2016). For breeding, linkage drag due to close linkage between favorable genes/alleles and genes with unfavorable alleles can hamper the transfer of the former genes to elite material. The recombination limitation due to sequence divergence and structural variation can also be an important bottleneck for introgression breeding strategies aiming to transfer resistance genes from wild relatives in crop’s genome. An improved knowledge of the synteny and collinearity between the different species, as well as of the recombination landscapes, is essential to optimize breeding schemes involving the different species of the *Citrus* genus.

Due to the high heterozygosity of citrus germplasm, most citrus genetic maps were developed from first-generation crosses, and segregation analyses allowed genetic maps to be developed for each of the parents and, in some cases, consensus genetic maps. The first high-density genetic map of citrus, based on codominant markers, was established in the framework of the project of the International Citrus Genome Consortium (ICGC) aiming to establish the citrus genome reference sequence from a haploid clementine (*C.* × *aurantium* var. *clementina* ined.; Wu et al., 2014). The clementine genetic map based on 961 SNP, SSR and Indel markers (Ollitrault et al., 2012) was used for the final assembly of the genome in pseudomolecules. High-density maps of sweet orange (*C. x aurantium* var. *sinensis*) with 943 markers (Xu et al., 2013) and mandarin with 706 markers (Shimada et al., 2014) were also published during the same period. More recently, the next generation sequencing method (NGS) combined with the reduction of genome complexity were used to produce medium- to high-density genetic maps. Guo et al. (2015) produced two pummelos maps from an F1 cross using RADSeq. DARTSeq was successfully applied to produce a synthetic map of Murcott [(*C.* x *aurantium* var. *sinensis*) x *C. reticulata*] and sweet orange (Curtolo et al., 2017a) as well as Sunki mandarin (*C. reticulata* var. *austera*) and Rubidoux trifoliate orange maps (Curtolo et al., 2017b). Genotyping by sequencing (GBS) allowed researchers to establish saturated genetic maps of trifoliate orange and sweet orange (Huang et al., 2018) as well as Ellendale tangor (*C.* × *aurantium* var. *tangerina ined.* X *C.* x *aurantium* var. *sinensis*) and Fortune mandarin (*C.* x *aurantium* var. *clementina* X (*C.* × *aurantium* var. *tangerina ined.* X *C.* x *aurantium* var. *paradisi*) (Ollitrault et al., 2021). The higher density integrated linkage map (4,163 markers) was published by Xu et al. (2021) from a clementine x trifoliate orange family using specific locus amplified fragment sequencing (SLAF-seq) technology. A few comparative genetic mapping studies analyzed the synteny and collinearity in cultivated citrus and trifoliate orange. The conservation of synteny was complete between sweet orange, pummelo and clementine, and the linear order of markers also appeared to be highly conserved between these species (Ollitrault et al., 2012). From partial genetic mapping, Bernet et al. (2010) reported high synteny and collinearity between Fortune mandarin, Chandler pummelo (*C. maxima*), sour orange and trifoliate orange. More recently, the availability of pseudo chromosome assemblies of citrus genomes allowed the comparison of genetic and physical maps and globally confirmed the good conservation of marker order between different cultivated citrus species (*C. reticulata*, *C.* x *aurantium* var. *sinensis* and *C.* x *aurantium* var. *clementina*) and *C. trifoliata* (Curtolo et al., 2017b; Huang et al., 2018; Xu et al., 2021).

The aim of the present work was to perform a large comparative mapping study that includes most of the main clades of the *Citrus* genus (**Figure 1**) and, particularly, the Oceanian clade considering its importance in breeding projects for HLB resistance. It concerns five species (*C. maxima, C. medica, C. reticulata, C. trifoliata* and *C. glauca*), two horticultural groups resulting from interspecific admixture (clementine, and lemon) and two recent interspecific hybrids: *C. australis* x *C. inodora* and *C. maxima* x *C. reticulata*. The recombination landscape and non-Mendelian segregation along the genome were analyzed. As a consequence of the high synteny and collinearity observed, we were able to establish a consensus map. It includes 10,756 loci (7,915 gene-based markers and 2,841 SNPs located-out gene sequences) and encompasses 1,005.3 cM. This consensus map was anchored on most of the published citrus genomes assembled in pseudomolecules. The synteny and the collinearity between our consensus genetic map and the different genome assemblies were analyzed.

## 2 Materials and methods

### 2.1 Progenies

Ten progenies were used to establish nine genetic maps (**Supplementary Table 1**) with a total of 1,216 hybrids analyzed by GBS. Individual maps were generally established from a single parental combination. However, the *C. reticulata* map was established using three progenies involving the same *C. reticulata* Cleopatra mandarin variety (Cleopatra mandarin x trifoliate orange, Cleopatra mandarin x Troyer citrange and Troyer citrange x Cleopatra mandarin). *C.* x *aurantium* var. *clementina* was implemented with two progenies (Chandler pummelo x Clementine and Clementine x Finger limes [*C. australasica*]). All progenies were diploid except for the Mediterranean lemon x “Giant Key” lime progenies that were triploid hybrids. Indeed, the Giant Key lime is tetraploid, resulting from chromosome doubling of the Mexican lime (*C.* x *aurantiifolia* var. *aurantiifolia*) (Ahmed et al., 2019). For all families, two replicates of each parent were included in the analysis.

### 2.2 GBS analysis

Library preparation: Genomic DNA was isolated using the Plant DNAeasy kit (Qiagen) according to the manufacturer’s instructions. The genomic DNA concentration of each sample was adjusted to 20 ng/μL, and *Ape*K I GBS libraries were prepared following the protocol described by Elshire et al. (2011) with 96 DNA samples multiplexed per the GBS library. 10 µL of each DNA sample (200 ng) were digested with the *Ape*K I enzyme (New England Biolabs, Hitchin, UK). Digestion took place at 75°C for 2 h. The ligation reaction was completed in the same plate as the digestion using the T4 DNA ligase enzyme (New England Biolabs, Hitchin, UK) at 22°C for 1 h. Then, the ligase was inactivated prior to pooling the samples by holding it at 65°C for 20 min. For each library, ligated samples were pooled and PCR amplified in a single tube. Genome complexity was reduced using PCR primers with one selective base (A) as described by Sonah et al. (2013).

Sequencing: For progenies 1 to 7, single-end (150 pb) sequencing was performed on two lanes of an Illumina HiSeq4000 platform at the Genoscope facilities (Paris, France). For progenies, 8 to 10 pair-end sequencing was performed on one lane per library of an Illumina HiSeq4000 platform at Genewiz facilities.

Variant calling: RAW sequencing data were cleaned with cutadapt (Martin, 2011) and demultiplexed with GBSX (Herten et al., 2015). SNP genotype calling was then performed with the VCF-Hunter 2.1.0 pipeline (https://github.com/SouthGreenPlatform/VcfHunter) as described in Baurens et al. (2019), using the Clementine v1.0 genome assembly (https://phytozome-next.jgi.doe.gov/info/Cclementina_v1_0) as the nuclear reference genome. Positions with less than 10 reads were considered as missing data. Polymorphic positions were filtered for diallelic SNPs and minor allele frequency greater than 0.05.

For progenies involving species widely different from the reference genome, we filtered to keep only those SNPs within annotated gene sequences. This reduced error rates and allowed for more accurate comparative mapping of shared marked genes. This strategy was applied for the *C. trifoliata*, *C. glauca* and *C. australis* x *C. australasica* maps, as well as for the *C.* x *aurantium* var. *clementina* map, for which an interspecific progeny with *C. australasica* was used in part. Only one SNP per gene was retained, selecting the one with the least amount of missing data. For the other progenies, a filter was made on a minimal distance of 5 kb between successive markers.

### 2.3 Genetic mapping

The two-way pseudo-testcross mapping strategy implemented for genetic mapping from progenies resulting from crosses between two heterozygous parents (Ritter et al., 1990) and used in previous high-density mapping studies in citrus (Ollitrault et al., 2012; Guo et al., 2015; Curtolo et al., 2017a; Huang et al., 2018) was applied to establish parental genetic maps. For each map, SNP markers were selected according to their respective heterozygosity for the mapped parent and homozygosity for the other one. Each set of data was filtered to retain markers and hybrids with less than 15% of missing data. The triploid progenies lemon x Giant Key lime was treated as a diploid one with heterozygous genotype X/Y attributed for both XXY and XYY allele doses. For the selected markers, heterozygous XY for lemon and homozygous YYYY for Giant Key, it allowed for an unambiguous inference to be made about the haploid lemon gamete from the genotyping data.

Linkage analysis and genetic mapping were then performed from the inferred gamete genotypes using JoinMap5 (https://www.kyazma.nl/index.php/JoinMap/). Linkage mapping was performed in the Hap option. Markers were grouped using the independence LOD score. Phases (coupling and repulsion) of the linked marker loci were automatically detected by the software. Map distances were estimated in centimorgan (cM) using the regression mapping algorithm and the Kosambi distance. After a first mapping round, singletons were identified. On the high-density maps, the probability of having two successive crossovers within a small genomic area is very low, whereas genotyping errors strongly affect the estimation of genetic distances that erroneously expand the genetic linkage groups. Therefore, as recommended by van Os et al. (2005), we replaced singletons with missing data using a homemade excel page routine and performed a second mapping round. At the same time, a few individuals displaying an aberrant number of recombination events (according to the distribution of the number of recombination events across all hybrids) were removed, because of possible poor quality genotype-calling.

### 2.4 Analysis of segregation distortion

The matrix of phased data resulting from each genetic map analysis was used to study the skewed segregation all along the genome. The p-values for the chi-square test according to a 0.5 theoretical frequency for each allele were computed with Excel, and we used the approach proposed by Benjamini and Hochberg (1995) to limit the false discovery rate (FDR) in multiple testing; the approach was performed according to the method of Storey (2002) with a q-value threshold of 0.05. The results were visualized in a Circos plot.

### 2.5 Analysis of recombination landscapes

The recombination landscape was estimated from the genetic position of the different genetic maps and the physical one in the Clementine v1.0 assembly, removing the markers displaying discrepancies for synteny and the ones of the misplaced and inverted area of the chr3 of the clementine assembly. Local recombination rates were estimated with MareyMap Online (Siberchicot et al., 2017) using the Loess function adjustment (wherein a two-degree polynomial is fitted in each sliding window) with a span parameter of 0.10. The results were visualized in a Circos plot.

### 2.6 Anchoring of genetic maps on published assemblies

The anchorage of the genetic maps on different published genome assemblies in pseudochromosomes was performed using the locOnRef tool of the Scaffhunter toolbox (Martin et al., 2016).

We analyzed the synteny and collinearity of our genetic maps with related published genome assemblies. The clementine genetic map was compared with the *C. clementina* V1.0 genome (Wu et al., 2014). For pummelo, the Chandler map was anchored in the *Citrus maxima* (*C. grandis*) genome v1.0 (Wang et al., 2017) and *Citrus maxima* Cupi Majiayou v1.0 genome (Lu et al., 2022). For the sweet orange, the representative maps of its two ancestral species *C. reticulata* (represented by Cleopatra mandarin) and *C. maxima* (represented by Chandler pummelo) and the *C. maxima* x *C. reticulata* hybrid (Pink x Tardia) were anchored on the following: (i) the first citrus genome assembly *Citrus sinensis* Valencia genome v1.0 (Xu et al., 2013), (ii) the *Citrus sinensis* Di-Haploid Sweet Orange (DHSO) v3.0 (Wang et al., 2021) and (iii) the recent haplotypes genome assembly *Citrus sinensis* cv. Valencia DVS_A genome v1.0 and *Citrus sinensis* cv. Valencia DVS_B genome v1.0 published by Wu et al. (2022). The *C. trifoliata* map was anchored on *C. trifoliata* v1.3.1 (Peng et al., 2020) and ASM1835013v1 (Huang et al., 2021). The C. lemon map was anchored on (i) the two haplotype assemblies of Di Guardo et al. (2021): *Citrus limon* L. Burm f. genome v1.0 – Primary, *Citrus limon* L. Burm f. genome v1.0 – Alternative and (ii) the two haplotypes chromosome-scale assembly of *Citrus limon* cv. Eureka genome v1.0 (Bao et al., 2023). The *C. australis* x *C. inodora* map was anchored on the *C. australis* genome v1.0 (Nakandala et al., 2023).

The consensus genetic map was anchored on all genome assemblies mentioned above plus the *C. japonica* var. *hindsii* S3y-45 genome v2.0 (Wang et al., 2022).

### 2.7 Synteny and collinearity

The synteny and collinearity of genetic maps with genome assemblies and between genetic maps were visualized using Circos (Krzywinski et al., 2009) in Galaxy (Rasche and Hiltemann, 2020) and drawing Marey maps using Excel. Collinearity was estimated with the Spearman’s rank correlation coefficient.

### 2.8 Consensus genetic maps

A composite map was constructed using LPmerge v1.7 (Endelman and Plomion, 2014) for each chromosome, choosing the linkage group with the least root mean-squared error (RMSE) over the “max.interval” parameter range (1–10) evaluated. Four parameters were tested: weighting for population size (N = 68-187), weighting for the number of markers (N = 987-4436), ratio between the number of markers and population size and an unweighting model. The map weighted by the number of markers presented the least RMSE among the max. interval tested, and it was chosen.

## 3 Results

### 3.1 Individual genetic maps and comparison with *C. clementina* v1.0 reference genome

The number of mapped SNPs for each parent varies from 760 for *C. medica* to 4,436 for the *C. maxima* x *C. reticulata* hybrid, with an average of 2,162.3 markers/map (**Table 1**; **Supplementary Table 2**). Nine linkage groups (LGs) corresponding to the nine chromosomes of the citrus haploid genome are found for most parents, with the exception of *C. medica* with 11 LGs (2 LGs for chr3 and chr6) and *C. trifoliata* (2 LGs for chr8). For *C. medica*, large regions of the genome appear to have no heterozygous markers; therefore, it is not possible to map them genetically. A similar situation, with a large genomic region in complete homozygosity in the center of chr8 of trifoliate orange, results in its division into two LGs. Overall, the maps of *C. medica*, *C. trifoliata* and *C. aurantium* var *clementina* show the most irregular coverage, with numerous gaps larger than 5 cM.

**Table 1:** Statistics of the nine individual genetic maps.

	n	LG	1	2	3	4	5	6	7	8	9	all
*C. maxima*	194	Mk	320	388	423	226	356	294	273	297	175	2752
		MS	99.61	97.31	124.31	79.98	81.55	69.68	81.57	102.17	102.27	838.46
		AGS	0.93	0.89	0.94	0.98	0.77	0.82	0.95	1.01	1.38	0.96
		BGS	4.13	4.67	3.25	4.02	2.58	2.58	4.65	3.13	5.35	5.35
		NGS5	0	0	0	0	0	0	0	0	1	1
		TGS5	0.00	0.00	0.00	0.00	0.00	0.00	0.00	0.00	5.35	5.35
		U P	108	110	133	83	105	84	87	102	75	887
*C. reticulata*	127	Mk	214	233	272	118	169	169	169	147	161	1652
		MS	110.68	113	159.41	101.89	109.02	76.64	92.54	94.18	90.1	947.45
		AGS	1.88	1.4	1.63	2.04	1.87	1.42	1.93	1.74	1.67	1.73
		BGS	8.3	6.24	5.96	7.15	6.33	3.95	5.95	7.64	5.61	8.3
		NGS5	6	1	1	3	2	0	4	1	2	20
		TGS5	37.51	6.24	5.96	19.58	12.57	0.00	22.55	7.64	10.69	122.74
		U P	60	82	99	51	59	55	49	55	55	565
*C. medica*	203	Mk	87	35	110 + 7	144	89	35 + 30	52	110	61	760
		MS	19.25	75.61	67.2 + 2.1	85.89	47	33.2 + 17.4	71.47	111.9	68.59	599.71
		AGS	0.687	3.601	1.308	1.101	1.27	1.632	2.465	1.963	2.54	1.841
		BGS	4.45	25.94	5.26	3.34	4.28	6.44	9.77	15.84	11.79	25.94
		NGS5	0	3	1	0	0	1	4	4	6	19
		TGS5	0.00	41.30	5.257 + 50	0.00	0.00	6.44 + 50	31.32	35.96	43.39	163.67 + 100
		U P	29	22	55	79	38	33	30	58	28	372
*C. maxima x * *C. reticulata*	152	Mk	539	622	784	481	501	215	486	336	472	4436
		MS	106.68	130.36	179.81	77.72	96.82	54.98	96.14	110.94	73.18	926.63
		AGS	0.79	0.81	1	0.93	0.85	1.45	0.9	1.32	0.81	0.98
		BGS	2.63	2.63	10.64	3.3	5.21	9.96	4.62	8.09	3.29	10.64
		NGS5	0	0	3	0	1	3	0	2	0	9
		TGS5	0.00	0.00	22.77	0.00	5.21	23.44	0.00	13.55	0.00	64.96
		U P	136	161	180	85	115	39	108	85	91	1000
*C. x aurantium var clementina*	187	Mk	92	114	198	125	131	106	55	84	82	987
		MS	115.99	132.43	193.98	115.4	155.03	91.39	86.31	131.34	75.63	1097.5
		AGS	2.47	2.28	1.83	1.46	1.78	1.79	2.78	2.68	1.61	2.08
		BGS	16.54	10.65	11.94	6.92	22.64	19.2	18.3	16.25	8.7	22.64
		NGS5	6	8	7	4	3	3	4	9	4	48
		TGS5	59.34	64.73	63.81	23.56	39.13	36.25	53.21	85.35	28.73	454.11
		U P	48	59	107	80	88	52	32	50	48	564
*C. x limon var limon*	108	Mk	426	518	670	430	389	361	408	372	344	3918
		MS	88.08	85.31	171.51	109.76	117.77	71.66	109.41	111.43	66.43	931.36
		AGS	1.01	1.09	1.08	1.14	1.09	1.17	1.14	1.43	1.13	1.14
		BGS	4.65	3.71	3.71	5.23	3.71	4.65	5.17	5.63	2.81	5.63
		NGS5	0	0	0	1	0	0	1	2	0	4
		TGS5	0.00	0.00	0.00	5.23	0.00	0.00	5.17	11.05	0.00	21.45
		U P	88	79	160	97	109	62	97	79	60	831
*C. trifoliata*	68	Mk	166	193	282	149	182	104	128	137	158	1499
		MS	141.82	138.53	191.68	116.62	129.8	93.52	118.07	47.44 + 54.72	118.22	1 150.44
		AGS	2.68	1.92	1.92	2.16	2.09	2.75	2.41	1.33	2.23	2.17
		BGS	10.45	5.91	5.95	5.91	7.41	11.53	8.92	4.43	6.78	11.53
		NGS5	6	2	4	4	2	4	5	4	3	34
		TGS5	45.77	11.48	23.11	22.72	13.32	30.64	35.27	33.78 + 50	18.60	234.68 + 50
		U P	54	73	101	55	63	35	50	42	54	527
*C. glauca*	173	Mk	175	248	318	230	195	139	221	160	160	1846
		MS	96.55	95.67	156.47	78.74	99.44	82.86	73.41	88.06	81.51	852.71
		AGS	1.18	0.99	1.09	0.89	1.18	1.34	0.86	1.38	1.24	1.13
		BGS	4.35	4.06	6.21	3.25	13.67	5.61	5.5	5.17	3.47	13.67
		NGS5	0	0	2	0	2	1	1	1	0	7
		TGS5	0.00	0.00	11.84	0.00	20.65	5.61	5.50	5.17	0.00	48.77
		U P	83	98	144	89	85	63	86	65	67	780
*C. australis x * *C. inodora*	171	Mk	211	244	367	250	202	190	181	188	186	2019
		MS	108.12	142.73	164.93	137.85	95.32	95.15	114	112.35	129.26	1099.71
		AGS	1.3	1.41	1.17	1.21	1.27	1.15	1.52	1.56	1.7	1.37
		BGS	3.84	3.96	3.63	3.67	7.17	4.15	5.86	4.46	6.59	7.17
		NGS5	0	0	0	0	1	0	2	0	3	6
		TGS5	0.00	0.00	0.00	0.00	7.17	0.00	11.29	0.00	17.42	35.89
		U P	84	102	142	115	76	84	76	73	77	829

n, number of hybrids; LG, linkage groups; Mk, number of markers; MS, Map size; AGS, Average gap size; BGS., Biggest gap size; NGS5; number of gap size > 5cM; TGS5; sum of gaps > 5cM; UP, Unique positions.

The nine genetic maps span from 599.7 cM for *C. medica* to 1,150.4 cM for *C. trifoliata*, with an average of 938.2. The number of unique positions by map varies between 372 for *C. medica* and 1,000 for the *C. maxima* x *C. reticulata* interspecific hybrid, with an average of 706.1 positions. The average gap size over individual maps varies between 0.96 cM for *C. maxima* and 2.17 cM for *C. trifoliata* (average over all maps: 1.5 cM), whereas the biggest gap size varies between 5.35 cM for *C. maxima* and 25.34 cM for *C. medica*.

The number of genes from the *Clementine* v1.0 assembly anchored by a mapped SNP marker varies respectively between 501 and 3,432 for *C. medica* and *C. maxima* x *C. reticulata* (**Supplementary Table 2**).

**Figure 2:**
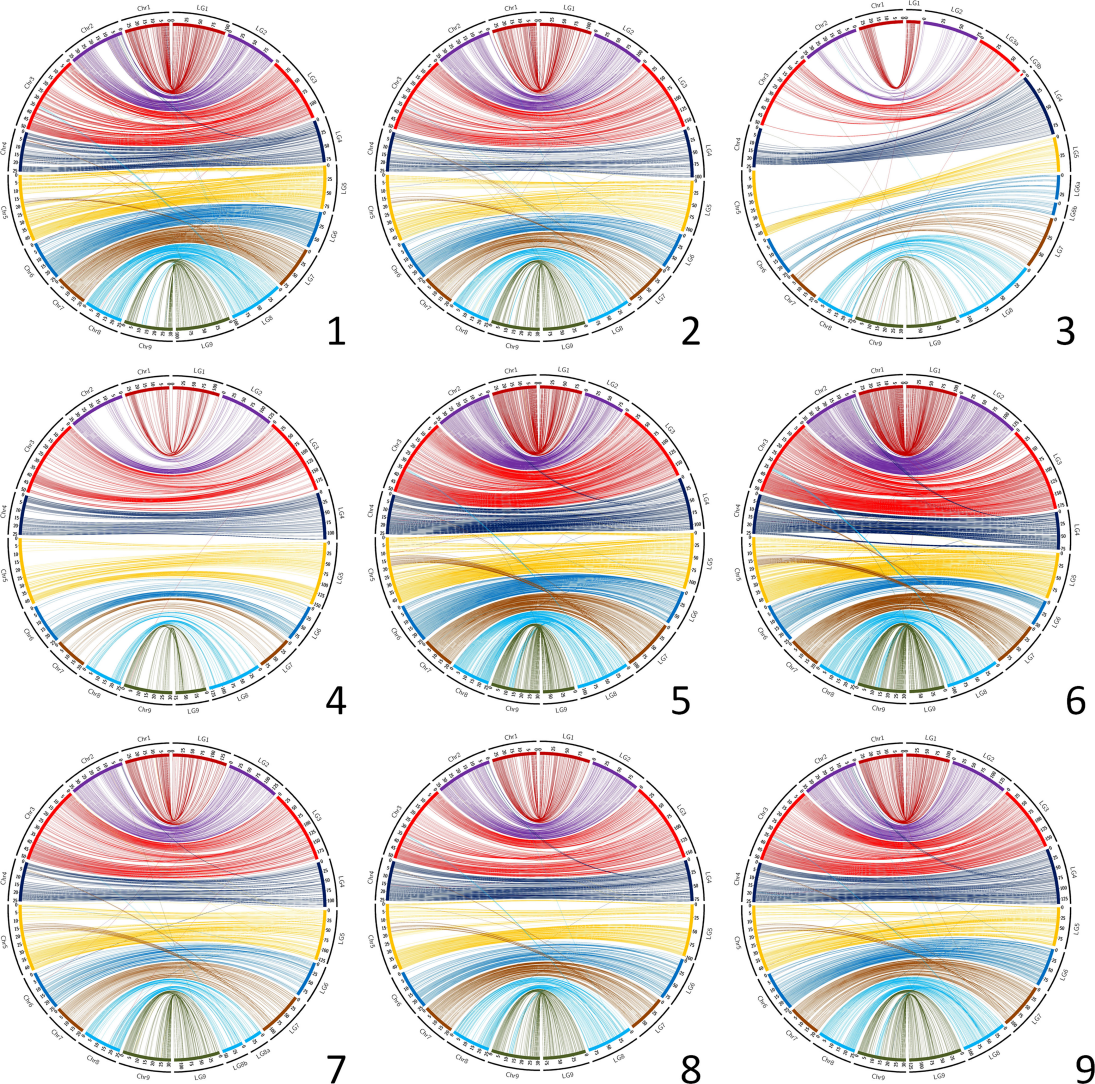
Link between the nine genetic maps and the *C. clementina* v1.0 genome assembly. Chr, *C. clementina* chromosomes; LG, linkage groups of the genetic maps. 1 (up/left): *C. maxima*; 2 (up/center): *C. reticulata*; 3 (up/right): *C. medica*; 4 (center/left): *C. x aurantium var clementina*; 5 (center/center): *C. limon var limon*; 6 (center/right): *C. maxima x C. reticulata*; 7 (down/left): *C. trifoliata*; 8 (down/center): *C: glauca*; 9 (down/right): *C. australis x C. inodora*.

Circos representations of the links between genetic positions and physical ones in the *C. clementina* v1.0 genome assembly (**Figure 2**) testify to a good coverage of the whole genome for most parents. However, as previously mentioned, the *C. medica* map displays very large gaps, and important gaps are also identified for *C. clementina*, particularly in chr2, chr5 and chr8, as well as in the middle region of chr4. The Circos representations (**Figure 2**) and Marey maps (**Supplementary Figure 1A**) for the other parents with high marker density reveal discrepancies between genetic maps and *C. clementina* assembly for several genomic regions. Most of these discrepancies are shared by all genetic maps. They are particularly clearly displayed for the *C. maxima* x *C. reticulata* parent that has the higher marker density (**Supplementary Figure 1B**). These systematic discrepancies concern the following: (i) genomic regions of chr5 (12.8-19.4 Mb) and chr4 (around 5 Mb) genetically mapped at the end of LG7; (ii) genomic regions of chr3 (around 35 Mb) and 9 (11.1-14.5 Mb) genetically mapped on the middle of LG8; (iii) a genomic region at the beginning of chr2 (0.7-4.2 Mb) mapping at the beginning of LG4; and a small genomic region of chr8 (around 16.5 Mb) mapping at the beginning of LG6. In addition, a misplaced and inverted region of 5.2 Mb is revealed by Marey representation (**Supplementary Figure 1**) in chr3 by the different genetic maps, including those of *C.* x *aurantium* var. *clementina* and *C. medica*.

Despite these small discrepancies, the global synteny of all our genetic maps with the *C. clementina* v1.0 assembly is high, with insignificant variations between the nine maps (from 0.961+/−0.019 to 0.997+/-0.026, respectively, for *C. maxima* x *C. reticulata* and clementine; **Supplementary Table 3**). The lowest mean values across the nine maps by linkage group for LG7, LG8 and LG4 were found to be consistent with our observations from the Marey maps and Circos representations. For the syntenic markers, the collinearity between the different genetic maps and the *C. clementina* v1.0 assembly is high, with the Spearman’s rank correlation varying between 0.935+/−0.054 and 0.997+/−0.001, respectively, for *C.* x *limon* var. *limon* and for *C. trifoliata* (**Supplementary Table 4**).

**Figure 3:**
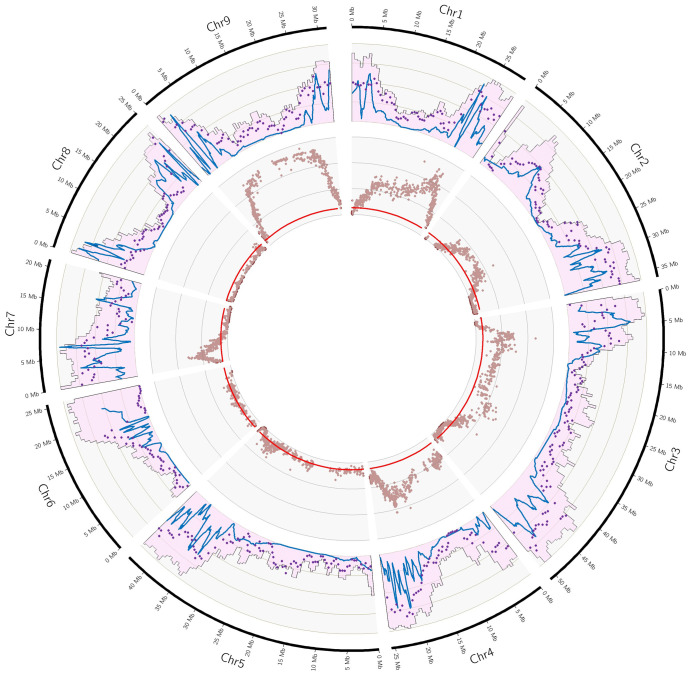
Gene and marker densities, recombination landscape and non-Mendelian segregations of the *C. maxima x C. reticulata* parent. External Outer ring: Pink histogram: gene density (scale 0-50%), blue line local recombination (scale 0-20 cM/Mb; purple dot: number of markers (scale: 0-5/100kb); inner ring: red line Threshold for Qvalue (0.05) significance; brown dot: Qvalue for Mendelian segregation (scale: 0-15).

Taking advantage of the good collinearity between the different maps and the *C. clementina* v1.0 genome, this genome assembly was used as a template to analyze and compare the distribution of skewed segregation and the recombination landscape of the different parents. It is based on syntenic markers of each map with the genome assembly. The probably misplaced region of chr3 of the clementine assembly was removed for these analyses. The distributions along the genome of mapped markers, skewed markers and the recombination landscape are synthetized in Circos plots (**Figure 3**; **Supplementary Figure 2**; **Table 2**).

**Table 2:** Skewed segregations.

	Chr.	1	2	3	4	5	6	7	8	9	Total
*C. reticulata*	Sk. nb	1	1	14	0	1	5	89	1	5	117
	%	0.5%	0.4%	5.1%	0.0%	0.6%	3.0%	52.7%	0.7%	3.1%	7.1%
*C. maxima*	Sk. nb	0	0	0	0	0	0	0	0	0	0
	%	0.0%	0.0%	0.0%	0.0%	0.0%	0.0%	0.0%	0.0%	0.0%	0.0%
*C. medica*	Sk. nb	87	6	4	1	1	13	34	10	1	157
	%	100.0%	17.1%	3.4%	0.7%	1.1%	20.0%	65.4%	9.1%	1.6%	20.7%
*Clementine*	Sk. nb	12	2	11	1	5	1	2	2	6	42
	%	13.0%	1.8%	5.6%	0.8%	3.8%	0.9%	3.6%	2.4%	7.3%	4.3%
*C. x limon*	Sk. nb	136	7	8	66	374	8	5	245	0	849
	%	31.9%	1.4%	1.2%	15.3%	96.1%	2.2%	1.2%	65.9%	0.0%	21.7%
*C. maxima x C. reticulata*	Sk. nb	508	267	453	481	276	55	260	7	469	2776
	%	94.6%	43.0%	57.9%	100.0%	55.1%	25.6%	53.5%	2.1%	99.4%	62.6%
*C. trifoliata*	Sk. nb	22	0	57	0	3	39	2	70	1	194
	%	13.3%	0.0%	20.2%	0.0%	1.6%	37.5%	1.6%	51.1%	0.6%	12.9%
*C. australis x C. inodora*	Sk. nb	102	95	141	37	83	19	64	64	23	628
	%	48.3%	38.9%	38.4%	14.8%	41.1%	10.0%	35.4%	34.0%	12.4%	31.1%
*C. glauca*	Sk. nb	0	13	30	4	0	1	7	0	84	139
	%	0.0%	5.2%	9.4%	1.7%	0.0%	0.7%	3.2%	0.0%	52.5%	7.5%

Sk.nb, number of significantly skewed markers; %, % of significantly skewed markers.

The recombination landscape was analyzed for all maps except for that of Corsican citron (*C. medica*), which was too incomplete. Similar patterns were observed for the other eight maps, with regions with low levels of recombination corresponding to low gene density regions and peaks of recombination in regions of high gene density (**Supplementary Figure 2**). Looking at the *C. maxima* x *C. reticulata* figure as an example (**Figure 3**), we observe regions of low recombination in chr1, chr2, chr3, 4 and chr5 corresponding to the lowest gene density regions of these different chromosomes. Large regions with no or very few recombinations are observed in chr6 (first 10 MB of the chromosome), chr8 (5 to 15 MB) and chr9 (5 to 20 Mb). Chr7 is the only one to display recombinations events all along the genome as well as no region with low gene density.

The segregation of *C. maxima* cv. Chandler (**Supplementary Figure 2A**) appears to be totally Mendelian, whereas limited distortions are observed for clementine (4.26%; **Supplementary Figure 2D**), *C. reticulata* cv Cleopatra (7.08%; **Supplementary Figure 2B**) and *C. glauca* (7.53%; **Supplementary Figure 2G**). For *C. reticulata* cv Cleopatra, most of the skewed markers are grouped in chr7, where an imbalance between alleles reached 0.7/0.3. A similar imbalance is observed for chr9 of *C. glauca* that contains 60.4% of the skewed markers for this parent (**Table 2**; **Supplementary Figure 2G**). For C. *trifoliata*, the global rate of skewed markers is 12.9% (**Supplementary Figure 2I**). No or very few (<2%) distortions were found in chr2, chr4, chr5, chr7 and chr9, whereas half of the markers have skewed segregation in chr8, reaching an imbalance of 0.94/0.06 for the two alleles. 20.7% of the markers display skewed segregation for *C. medica* (**Supplementary Figure 2C**). They are located mostly in chr1 and chr7. In chr7, the distortion reaches the almost complete elimination of one haplotype. For *C. x limon*, 96.1%, 65.9%, 31.9% and 15.4% of the markers were skewed respectively for chr5, chr8, chr1 and chr4, with a global rate of distortion of 21.7% (**Supplementary Figure 2E**). The higher intensity of distortion is observed for chr5, where it reaches an imbalance of 0.8/0.2 between the two alleles. The rate of skewed markers for *C. australis* x *C. inodora* reaches 31.1% with a distribution across all chromosomes (**Supplementary Figure 2H**), and a maximum imbalance between alleles of around 0.7/0.3. C*. maxima x C. reticulata* segregation displays the higher level of skewed segregation with 62.7% of the markers. Almost all markers of chr1, chr4 and chr9; around half of chr2, chr5 and chr7; and 25.6% of chr6 display skewed segregations (**Table 2**; **Figure 3**). The level of distortion reaches imbalances of 0.85/0.15 in chr1 and chr9; 0.75/0.25 in chr3, chr4 and chr7; 0.7/0.3 in chr2; and 0.6/0.4 in chr6. We did not observe evidence for complete elimination of one allele. If we consider that the distortions result from unfavorable genes located close to the position with higher –log(Qvalue), we can observe that the evolution of the –log(Qvalue) pattern along the chromosome is closely linked with the segregation landscape (**Figure 3**). For chr1, the implied gene should be close to 25 Mb in a region with a high level of recombination. It results in a decreased distortion on both sides, leading to Mendelian segregation at the right end of the chromosome. On the left side, the decrease of the distortion is more limited in the 8-15 Mb region, which displays limited recombination and gene density. Then, from 8 Mb to the start of the chromosome (corresponding to high gene density and recombination rate), there is a strong decrease in distortion until reaching Mendelian segregation. For chr9, we can hypothesize that the unfavorable gene is located in the large region with very few recombination events and that its adverse effect influences segregation throughout this region. Its effect then decreases in the outer region, in line with high recombination rates and gene density. For chr7, the unfavorable gene may be located around 2.5 Mb in a region with a high recombination rate, and the maintenance of recombination all across the chromosome allows for recovery of Mendelian segregation on both sides. Similar interpretation can be made for the profile of distortions in chr2, chr3 and chr4.

### 3.2 Relation of the genetic maps with corresponding published genome assembly in pseudo-chromosomes

The Circos analyses of the link between our genetic maps and the different genome assemblies (**Supplementary Figure 3**) reveal two forms of chromosome numbering. The genomes published by the International citrus genome consortium and USA research groups (*C. clementina* v1.0, TrO-USA, Swo-USA-HapA; Swo-USA-HapB), as well as the trifoliate orange published by Huazhong Agricultural University (TrO-China; (Huang et al., 2021), follow the numbering of *C. clementina* v1.0 that we used as a reference for our genetic mapping. The genomes published by the Chinese groups (Swo-China-V3, Pum-ChinaV1, Pum-ChinaV2, Lemon-China-HapA, and Lemon China-HapB), Italian groups (Lemon-It-Prim and Lemon-It-Alt) and Australian groups (*Australis*) adopt the numbering of the Swo-China-V1 (Xu et al., 2013). The correspondences between the numbering of our genetic maps and considered genome assembly are provided in **Supplementary Table 5**. The analyses of synteny and collinearity account for these correspondences.

For the *C. clementina* v1.0 genome, the synteny with the clementine genetic map is high (0.997) despite the apparently misplaced regions of the *Clementina* v1.0. It can be explained because the concerned regions are not covered by the genetic map due to full homozygosity in these areas. The collinearity is very high for chr1, chr2, chr3, chr4, chr8 and chr9, but the Spearman’s coefficient falls to 0.779 in chr6. It results in an average collinearity of 0.935+/−0.054.

For pummelo genomes, the synteny with the *C. maxima* cv. Chandler map is relatively low (0.887) with Pum-China-V1 due to numerous clusters of markers of the different chromosomes assigned in different linked groups. The synteny is high with the Pum-China-V2 assembly (0.987). For syntenic markers, collinearity with the genetic map is high for all chromosomes of both genome assemblies, except for chr9 of the Pum-China-V2, which has a Spearman’s coefficient value of 0.503. This is due to an inversion affecting half of the assembled pseudo-chromosome.

For lemons, we observed low synteny of the *C. x limon* genetic maps with Lemon-It-Prim and lemon-It-Alt (0.889 and 0.895, respectively). However, it is very high with the two haplotypes of Eureka lemon (0.996 and 0.994, respectively for Lemon-China-HapA and Lemon-China-HapB). The collinearity of the *C. x limon* genetic map is low with the two haplotypes of Lemon-It (0.839+/−0.124 and 0.848+/−0.129, respectively), with a particularly low value in chr4 (**Supplementary Table 6**), whereas it is high with the two Eureka lemon haplotypes (0.997+/−0.001 and 0.996+/−0.001).

The SwO-China-V3 displays very clear increases of synteny and collinearity with *C. reticulata*, *C. maxima* and *C. maxima* x *C. reticulata* genetic maps when compared with SwO-China-V1 (**Table 3**). Very high synteny values are observed for all chromosomes with SwO-USA-HapB. Similar results are observed with SwO-HapA, except for chr1 and chr9, displaying a potential reciprocal translocation (**Supplementary Figure 3**). The collinearity between the three considered genetic maps and the two sweet orange haplotypes assemblies is also very high for all SwO-USA-HapB chromosomes and chr2, chr3, chr4, chr5, chr6, chr7 and chr8 of SwO-USA-HapA.

**Table 3:** Synteny and collinearity between some genome assemblies and the related genetic maps.

Genome	Genetic map	nb markers	Synteny	Global Col.
C. clementina V1.0	*C. x aurantium var clementina*	987	0.997	0.935+/-0.054
Pum-China-V1	*C. maxima*	2647	0.887	0.998+/-0.000
Pum-China-V2	*C. maxima*	2571	0.987	0.932+/-0.105
Swo-China-V1	*C. maxima*	2324	0.909	0.983+/-0.022
	*C. reticulata*	1384	0.909	0.989+/-0.009
	*C. maxima x C. reticulata*	3696	0.921	0.969+/-0.035
Swo-China-V3	*C. maxima*	2705	0.984	0.998+/-0.000
	*C. reticulata*	1636	0.983	0.959+/-0.016
	*C. maxima x C. reticulata*	4395	0.995	0.975+/-0.020
SWO-USA-A	*C. maxima*	2697	0.898	0.998+/-0.001
	*C. reticulata*	1620	0.885	0.998+/-0.000
	*C. maxima x C. reticulata*	4363	0.870	0.996+/-0.002
SWO-USA-B	*C. maxima*	2709	0.985	0.999+/-0.000
	*C. reticulata*	1631	0.986	0.998+/-0.001
	*C. maxima x C. reticulata*	4393	0.995	0.997+/-0.002
Lemon-It-prim	*C. x limon*	3540	0.889	0.839+/-0.124
Lemon-It-alt	*C. x limon*	3448	0.895	0.848+/-0.129
Lemon-China-HapB	*C. x limon*	3880	0.996	0.997+/-0.001
Lemon-China-HapA	*C. x limon*	3876	0.994	0.996+/-0.001
TrO-USA	*C. trifoliata*	1484	0.991	0.969+/-0.014
TrO-China	*C. trifoliata*	1399	0.976	0.990+/-0.003
C. australis	*C. australis x C.inodora*	1987	0.993	0.998+/-0.001

Nb markers, number of markers linking the considered genome and genetic map; Global Col., average collinearity over the nine chromosomes.

The TrO-USA assembly displays very high synteny and collinearity with the *C. trifoliata* genetic map (0.991 and 0.969+/−0.014, respectively). For TrO-China, the synteny with the genetic map is a bit lower (0.976), mainly due to a cluster of markers of LG7 positioned in chr5 in the TrO-China assembly (**Supplementary Figure 3**). The collinearity of syntenic markers is high for both trifoliate orange genome assemblies but a little higher for TrO-China (0.990+/−0.003 versus 0.969+/−0.014 for TrO-USA).

The *C. australis* genome assembly displays very high synteny (0.992) and collinearity (0.998+/−0.001) with the *C. australis* x *C. inodora* genetic map.

### 3.3 Consensus genetic map and its comparison with individual genetic maps

To estimate the synteny and collinearity between the different genetic maps, we analyzed the links between the position on the *C. maxima x C. reticulata* map (that displays the higher marker density) and the eight other parental maps. The genes of *C. clementina* v1.0 were considered as markers to establish the link between genetic maps. Both synteny and collinearity estimated using the Spearman’s coefficient are higher than 0.992 for all genetic maps (**Table 4**). The Circos representations (**Supplementary Figure 4**) show that un-syntenic markers are dispersed over the map, and we did not observe any cluster of non-syntenic markers.

**Table 4:** Synteny and average collinearity over the nine chromosomes between Pink x Tardia genetic map and the eight other genetic maps.

	Nb Mark.	Synteny	Collinearity
*C. maxima*	655	0.995	0.995+/-0.003
*C. reticulata*	478	0.998	0.996+/-0.002
*C. medica*	164	1.000	0.993+/-0.002
*C. x aurantium* var. *clementina*	636	0.998	0.994+/-0.005
*C. x limon* var. *limon*	1328	0.999	0.992+/-0.009
*C. trifoliata*	559	0.995	0.993+/-0.004
*C. glauca*	703	0.994	0.992+/-0.004
*C. australis x C. inodora*	795	0.992	0.996+/-0.003

Nb Mark., number of markers linking the Pink x Tardia and the other genetic maps.

Given the high degree of synteny and collinearity observed, it was appropriate to draw up a consensus genetic map. Considering the very incomplete coverage of the whole genome by the *C. medica* genetic map, we established the consensus genetic map with the eight other available individual maps. We also excluded the *C. trifoliata* map for the implementation of the LG8 consensus map because the *C. trifoliata* chr8 corresponded with two unlinked LGs. The markers of the eight maps positioned in the same gene of the *C.* x *aurantium* var. *clementina* genome assembly were considered to be a single “gene marker” to establish the consensus genetic map. The consensus genetic map spans 1,005.27 cM and includes 10756 markers (7915 C. *clementina* “gene markers” and 2841 “non-genic markers”; **Table 5**; **Supplementary Table 7**). The size of the nine linkage groups varies from 90.10 cM for LG9 to 166.94 CM for LG3. The total number of unique positions is 2,808 with an average gap size of 0.36 similar for all chromosomes. Respectively, 95.35% and 99.92% of the gaps between unique positions are less than 1 cM and 4 cM (**Supplementary Figure 4**). Only two gaps are over 4 cM. Both are located in LG6: one at the start (5.65 cM) and one at the end (8.3 cM).

**Table 5:** Statistics of the consensus map.

LG	GM	NGM	TM	size (cM)	AGS	BGS	NGS5	TGS5	UP
1	873	292	1165	113.033	0.3	3.334	0	0.00	381
2	1062	330	1392	115.214	0.31	1.547	0	0.00	367
3	1404	457	1861	166.936	0.39	2.586	0	0.00	424
4	883	269	1152	104.76	0.4	3.714	0	0.00	263
5	851	394	1245	110.079	0.34	2.233	0	0.00	321
6	688	235	923	93.521	0.39	8.295	2	13.94	242
7	759	327	1086	98.811	0.34	3.138	0	0.00	290
8	670	280	950	112.813	0.38	2.592	0	0.00	297
9	725	257	982	90.098	0.41	1.63	0	0.00	223
all	7915	2841	10756	1005.265	0.36	8.295	2	13.94	2808

LG, linkage group; GM, gene markers; NGM, non-genic markers; TM, total markers; AGS, average gap size; BGS, biggest gap size; NGS5, number of gap size > 5cM; TGS5, sum of gaps > 5cM; UP, unique positions.

The 10,756 gene markers and non-genic markers correspond to 17,745 SNP markers of the initial individual maps. The number of markers shared by the consensus map and the individual ones varies from 985 for *C.* x *aurantium* var. *clementina* to 4,435 for *C. maxima* x *C. reticulata* (**Supplementary Table 8A**), and the collinearity between the consensus map and all individual maps is very high (between 99,79% for *C. limon* and 99,94% for *C. reticulata*; **Supplementary Table 8B**).

### 3.4 Anchoring of the consensus genetic map on different genome assemblies in pseudochromosomes

Among the 17,745 SNPs of the individual map anchored on the consensus one, 17,718 are located on the nine chromosomes of the *C. clementina* v1.0 assembly. Probes of 100 bases on each side of these 17,718 SNPs were defined from the *C. clementina* v1.0 sequence (**Supplementary Tables 9**, **10**) and anchored in 14 genome assemblies in pseudochromosomes in order to perform synteny and collinearity analysis. The rate of successful anchorage (**Table 6**) varies between 91.57% for TrO-China (Huang et al., 2021) and 99.18% for SwO-China-V3 (Wang et al., 2021). It is less than 94% for the two haplotypes of the lemon assemblies published in 2021 (Guardo et al., 2021). Interestingly, very good anchorage (>98%) is observed for the Australian species genome assemblies. For each genome assembly, one single marker by gene of the considered genome assembly was selected for synteny and collinearity analysis. The number of gene tags varies from 5,884 to 8,086, respectively, for Lemon-Italy-Alt and SwO-USA-B (**Table 6**). The detailed links between the consensus genetic map and the anchored genes for each genome are given in **Supplementary Table 11**. For the anchorage rates and the number of tagged genes, important differences are observed between the two haplotypes of lemons published in 2021 (Guardo et al., 2021) and the ones published in 2023 from PACBIO HIFi data (Bao et al., 2023), with a strong increase for the last ones. Similar improvement is observed between the first *C. sinensis* genome assembly and the more recent ones (Wang et al., 2021; Wu et al., 2022). The trifoliate orange assembly published in 2021 (Huang et al., 2021) displays lower tagging values than that released in 2020 (Peng et al., 2020).

**Table 6:** Statistics for the anchorage of the consensus genetic map on 15 genome assemblies.

Genome	NAP	GM	Synt.	Col.
Clementine-ICGC	17718	7840	0.976	0.997+/-0.002
SwO-China-V1	17117	6589	0.928	0.987+/-0.015
SwO-China-V3	17573	7803	0.998	0.998+/-0.001
SwO-USA-A	17472	7712	0.902	0.998+/-0.001 *
SwO-USA-B	17540	8086	0.998	0.998+/-0.001
Lemon-Italy-prim	16586	5938	0.890	0.870+/-0.100
Lemon-Italy-alt	16423	5884	0.895	0.874+/-0.100
Lemon-China-A	17556	7496	0.998	0.998+/-0.001
Lemon-China-B	17485	7438	0.998	0.995+/-0.003
TrO-USA	17321	7766	0.998	0.998+/-0.001
TrO-China	16224	5996	0.985	0.996+/-0.003
Kumquat-China	17197	7555	0.998	0.998+/-0.001
C.australis- Australia	17372	7166	0.996	0.998+/-0.001
Pummelo-ChinaV1	17455	7401	0.906	0.997+/-0.001
Pummelo-ChinaV2	16979	6855	0.998	0.939+/-0.111

NAP, Number of anchored probes; GM, number of gene markers; Synt, Synteny; Col., average collinearity over the nine chromosomes; * the collinearity was estimated for chromosome 2 to 8 due to the reciprocal translocation between chromosomes 1 and 9 of Valencia sweet orange.

The synteny between the consensus map and the different genome assemblies varies between 0.890 and 0.998 (**Table 6**). It is over 0.996 for eight of the assemblies, including SwO-China-V2 (**Figure 4.2**), SwO-USA-HAPB (**Figure 4.4**), Pum-China-V2 (**Figure 5.2**), *C. hindsii* (**Figure 5.4**), Lemon-China HapA and HapB (**Figures 6.3**, **6.4**), TrO-USA (**Figure 7.1**) and *C. australis* (**Figure 7.3**). The Circos plots reveal different kinds of pictures for the assemblies with lower values of synteny. The assemblies of SWO-China v1.0 (**Figure 4.1**), Pum-China-V1 (**Figure 5.1**), Lemon-It-prim (**Figure 6.1**), Lemon-It-alt (**Figure 6.2**) display numerous small genomic regions and isolated markers assigned to different linkage groups of the consensus genetic map. Most of these discrepancies with the consensus map are common in the comparison with the two lemon haplotypes and the Pum-China v1.0 assemblies. For the *C. clementina* v1.0 assembly (**Figure 5.3**) five small genomic regions have different locations on the consensus map. The lower global synteny of TrO-China (**Figure 7.2**) is principally caused by a 5 Mb genomic region of chr5 located in the LG7 of the consensus map. Finally, the low syntenic value (0.902) with the haplotype A of the Valencia sweet orange assembly (Wu et al., 2022) (**Figure 4.3**) results from a reciprocal exchange of genomic regions between chr1 and chr9, whereas synteny is very high for the others chromosomes.

**Figure 4:**
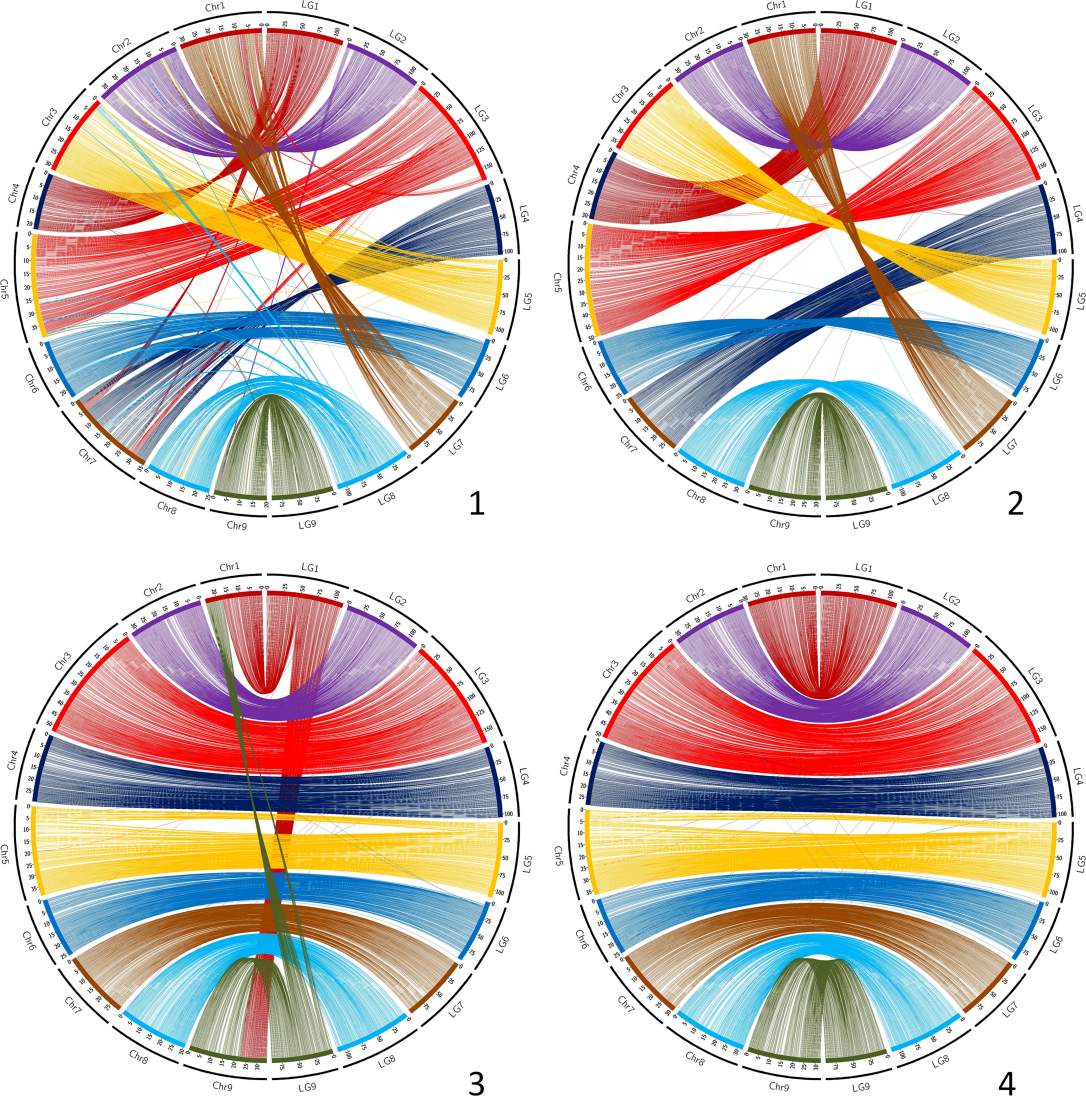
Link between the Consensus genetic map and genome assemblies. 1 (up/left): SwO-China-V1; 2 (up/right): SwO-China-V3; 3 (down/left): SwO-USA-HapA; 4 (down/right): SwO-USA-HapB. LG, linkage groups of the consensus genetic map; Chr, chromosome of the genome assembly.

**Figure 5:**
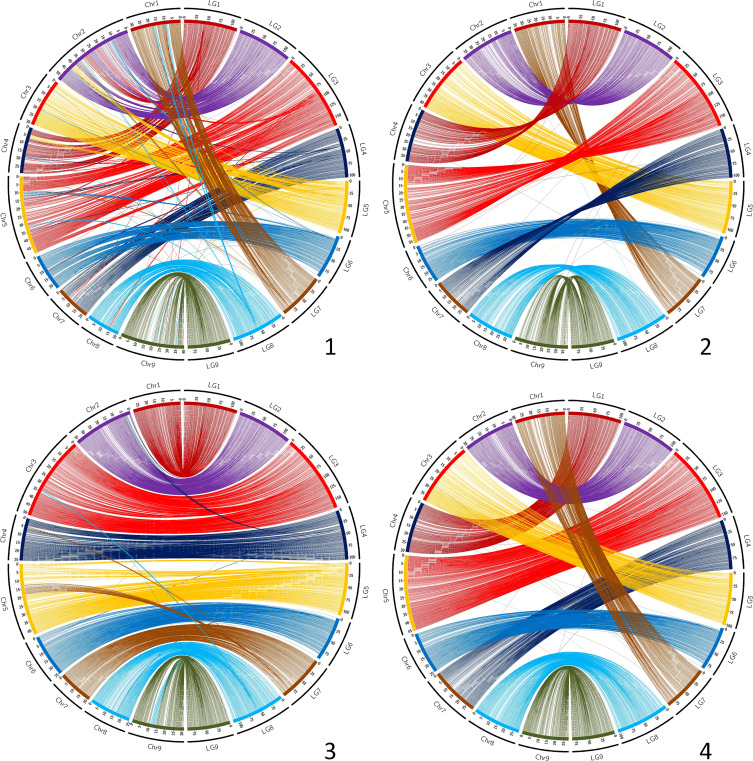
Link between the Consensus genetic map and genome assemblies. 1 (up/left): Pum-China-V1; 2 (up/right): Pum-China-V2; 3 (down/left): *C. clementina* V1.0; 4 (down/right): *C. hindsii.* LG, linkage groups of the consensus genetic map; Chr, chromosome of the genome assembly.

**Figure 6:**
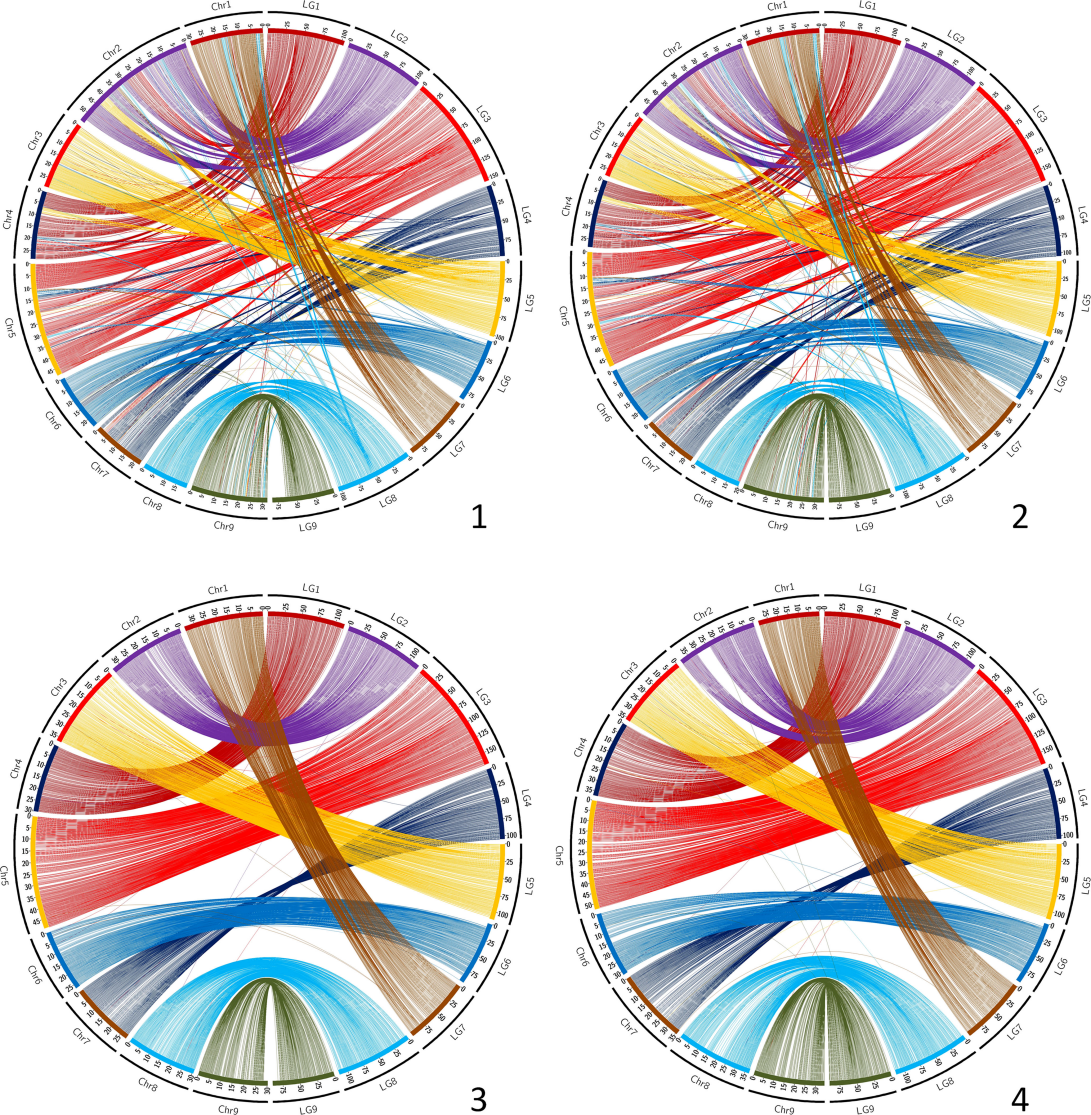
Link between the Consensus genetic map and genome assemblies. 1 (up/left): Lemon-It-prim; 2 (up/right): Lemon-It-alt; 3 (down/left): Lemon-China-HapA; 4 (down/right): Lemon-China-HapB. LG, linkage groups of the consensus genetic map; Chr, chromosome of the genome assembly.

**Figure 7:**
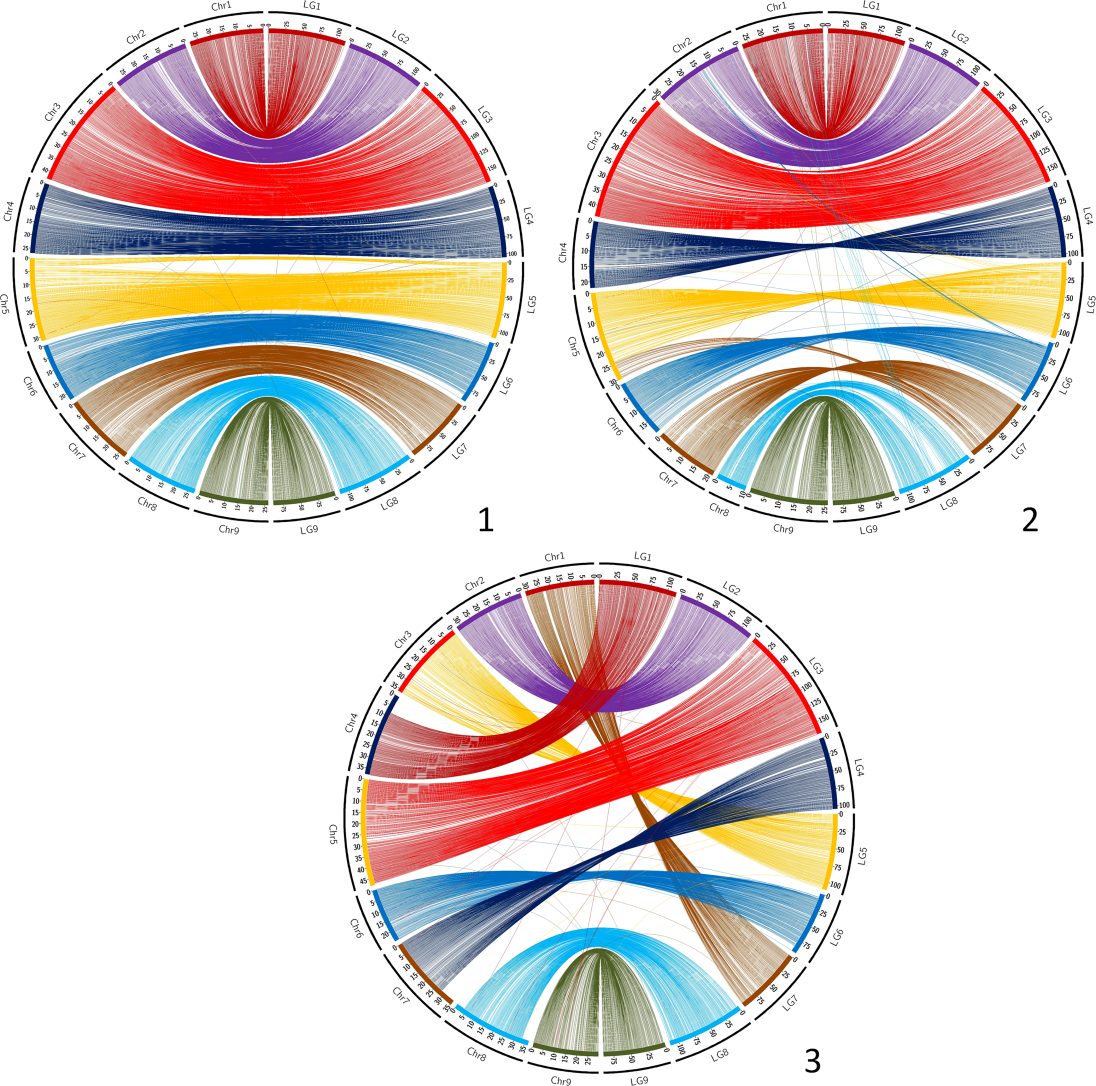
Link between the Consensus genetic map and genome assemblies. 1 (up/left): TrO-USA; 2 (up/right): TrO-China; 3 (down): *C. australis.* LG, linkage groups of the consensus genetic map; Chr, chromosome of the genome assembly.

The collinearity with the consensus map estimated by the Spearman coefficient is very high (> 0.995) with 11 genome assemblies (**Table 6**; **Supplementary Table 12**). It is a little lower for SwO-China-V1, but still high (0.987). The relatively low global value for Pummelo-ChinaV2 (0.939) is due to a displaced/inverted region in chr9, while collinearity is very high with all other chromosomes (0.996). The lower values (respectively 0.870 and 0.874) are observed for Lemon-It-Prim and Lemon-It-Alt with particularly low values (<0.7) for chr7 and chr8.

The synteny and collinearity of the consensus map with the *C. australis* assembly are very high (Nakandala et al., 2023). However, it is the only analyzed genome assembly for which genomic regions of several Mb are not anchored by the markers of the consensus genetic map. It is the case for 8 Mb at the beginning of chr4 (LG1), 7 Mb at the end of chr7 (LG4) and around 5 Mb at the beginning and end of chr8 (LG8).

## 4 Discussion

### 4.1 GBS using ApeK I as restriction enzyme is powerful for comparative genetic mapping and integration of genetic maps at the interspecific level

Numerous genetic maps were developed in citrus to explore the genetic control of several useful agronomic traits. However, initially, their comparison was hampered because most of them were established with dominant markers such as RAPD and AFLP. Multiallelic SSR markers allowed the first analyses of comparative genetic mapping to be conducted (Chen et al., 2008; Bernet et al., 2010; Ollitrault et al., 2012) but were limited by the relative low density of markers shared by the different maps. Shimada et al. (2014) proposed a gene-based marker approach to develop a framework genetic map, including 706 loci. This framework genetic map was useful to integrate other genetic maps based on common mapped genes. The same authors, also proposed that it should help to understand the regulation of gene expression by combining information on genetic loci and transcription profiles. However, the number of tagged genes and the resolution of their genetic map remained limited. Taking advantage of NGS, during the last 10 years, several medium- to high-density genetic maps of various *Citrus* species were produced using GBS (Huang et al., 2018; Ollitrault et al., 2021), RADSeq (Guo et al., 2015), DART-Seq (Curtolo et al., 2017a, 2017b) and SLAF-seq (Xu et al., 2021). However, it remained difficult to integrate the maps obtained with different species because they shared very few common SNP markers. In the present work, we successfully combined the preferential distribution of the *Ape*K I enzyme restriction site on coding regions, demonstrated in maize and barley (Elshire et al., 2011), soybean (Sonah et al., 2013), *Populus* (Schilling et al., 2014) and citrus (Oueslati et al., 2017; Ahmed et al., 2019), and the concept of gene-based markers to develop a high-density consensus genetic map. It was established from an individual map of three Asian ancestral species (*C. maxima*, *C. reticulata* and *C. trifoliata*), one Australian species (*C. glauca*), two Asian secondary species (*C.* x *limon* var. *limon* and *C.* x *aurantium* var. *clementina*), one F1 Asian interspecific hybrid (*C. maxima* x *C. reticulata*) and one Australian interspecific F1 hybrid (*C. australis* x *C. inodora*). Most of the individual genetic maps displayed a regular coverage of the citrus genome. However, the *C. medica* map displays very large gaps corresponding to the full absence of heterozygous markers in large genomic regions. This result can be explained by the origin of the Corsican citron (used as the *C. medica* representative), from a self-fecundation of the “Poncire commun” variety (Luro et al., 2012). Similarly, several genomic regions in chr2, chr5 and chr8 were not covered by the *C.* x *aurantium* var. *clementina* genetic map. This was also observed for the first reference clementine genetic map (Ollitrault et al., 2012) and was attributed to fully homozygous regions resulting from inbreeding in the origin of Clementine (Wu et al., 2014). Due to its incompleteness, the *C. medica* map was not used to establish the consensus genetic map. The consensus map spans 1,005.27 cM and encompasses 10,756 loci, including 7,915 gene-based markers and 2,841 SNPs located-out gene sequences. It presents 2,808 unique positions with an average gap size of 0.36 cM. The consensus genetic map shows much more regular genome coverage than some of the individual maps, where homozygosity due to inbreeding resulted in large gaps with no genome marking. Thus, only two gaps of more than 5 cM are observed for the consensus map (start an end of linkage group 6). The synteny and collinearity studies carried out between the consensus map and the different genome assemblies are therefore based on a much better representation of the whole genome than those carried out with the individual maps.

### 4.2 Comparative genetic mapping reveals a high synteny and collinearity between true citrus species and a similar recombination landscape

Previous comparative genetic mapping studies based on SSR markers suggested important synteny and collinearity between several cultivated citrus species [mandarin, clementine, pummelo, sweet and sour orange (Bernet et al., 2010; Ollitrault et al., 2012; Yu et al., 2016)] and even between cultivated citrus species (pummelo, sweet and sour orange) and *C. trifoliata* (Chen et al., 2008; Bernet et al., 2010). However, these conclusions were based on partial maps and low numbers of markers with a maximum of 418 markers shared between sweet orange and clementine (Ollitrault et al., 2012). The anchoring on the same reference genome of different high-density genetic maps, established with SNP markers (Yu et al., 2016; Huang et al., 2018; Ollitrault et al., 2021), highlighted the high synteny between genetic maps, even though they also evidenced some discrepancies between the considered genome assemblies and genetic maps. In the present work, the anchoring of the nine genetic maps on the *C. clementina* v1.0 genome assembly and the comparison between the *C. maxima* x *C. reticulata* map and the other eight individual genetic maps based on common gene-based markers revealed a very high synteny and collinearity for all genetically mapped parents. The conclusions for a highly conserved structure of the nuclear genomes therefore concern all the major clades of the true citrus group as defined by Swingle and Reece (Swingle and Reece, 1967), now joined in the new definition of the *Citrus* genus (Mabberley, 1998, 2022; Ollitrault et al., 2020). Our evidences from comparative genetic mapping are fully in agreement with the complete absence of large inter-chromosomal rearrangements between six species of the true citrus (*C. maxima, C. reticulata, C. medica, C. mangshanensis, C. trifoliata* and *C. australasica*) revealed by chromosome-specific painting (He et al., 2020). Therefore, the genome structure of the true citrus species appears to be globally highly conserved. Our conclusions are based on the ordering of 7915 genes, i.e. around 1/4 of all the genes distributed throughout the citrus genome. They do not presume the absence of moderate-sized structural rearrangements, particularly in gene-poor regions.

Taking advantage of this high synteny and collinearity, we analyzed the distribution of non-Mendelian segregation and the recombination landscape along the genome using the anchorage of the different genetic maps in the same reference (i.e., *C. clementina* v1.0 used for the genotype calling from GBS data). The genetic map size of the eight maps with good global coverage of the *C. clementina* v1.0 genome assembly (excluding *C. medica*) varies respectively between 852.3 and 1,099.7 cM for *C. glauca* and *C. australis* x *C. inodora*. In tomato, it has been proposed that sequence divergence at the interspecific level has an inhibitory effect on sexual recombination (Liharska et al., 1996; Chetelat et al., 2000). The map size variations observed between our different parents, which include pure species, F1 interspecific hybrids and complex admixture genomes, do not reveal such an effect in citrus. The recombination landscape along the genome was analyzed for the eight maps with good global coverage of the *C. clementina* v1.0 genome assembly. This landscape was similar and highlighted low/no recombination regions in genomic areas with very low gene density of chr1, chr2, chr3, chr4 and chr5, where centromeres were located by half tetrad analysis (Aleza et al., 2015). Very large regions of chr6, chr8 and chr9 with very low recombination levels were also revealed. These last regions include the centromere location (Aleza et al., 2015) but also encompass numerous gene sequences. Chr7 is the only one displaying recombination all throughout. For sexual breeding schemes, the revealed pattern of recombination will be very useful to optimize the management of multi-loci selection of genes located on the same chromosome.

Deviations from Mendelian segregation have been frequently described in citrus, particularly for the male parent markers (Carlos de Oliveira et al., 2007; Bernet et al., 2010; Ollitrault et al., 2012, 2021; Yu et al., 2016), probably due to pollen competition (Ollitrault et al., 2012). We also noticed the higher rates of skewed segregations for the male parents *C. australis* x *C. inodora* and *C. maxima* x *C. reticulata*. However, the female *C.* x *lemon* parent displays the third highest rate of skewed markers. The lower rates of non-Mendelian segregation were observed for several pure species (*C. maxima* cv Chandler, *C. reticulata* cv Cleopatra and *C. trifoliata*) used as male or female parents. Therefore, it is probable that various factors (e.g., sex, phylogenomic structure, parental combination) may affect Mendelian segregation. Recessive unfavorable mutations can result in strong gametophytic selection and therefore important deviation from Mendelian segregations. For *C. maxima* x *C. reticulata* (Pink pummelo x Tardia mandarin), the direct relation between the extent of skewed regions and recombination landscape is a good illustration of the impact of the variation of recombination rates along the genome and the extent of linkage drag. In the case of Corsican citron (*C. medica*) used as a pollinator, we observed a quasi-complete elimination of one haplotype at the beginning of chr7, where one S locus is located for gametophytic incompatibility in citrus, based on a S-RNase system (Liang et al., 2020; Ollitrault et al., 2021). This skewed segregation is similar to that occurring in reciprocal crosses between two self-incompatible varieties (Fortune mandarin and Ellendale tangor) sharing one incompatible allele at this S locus (Ollitrault et al., 2021). This suggests that Corsican citron may share with Chandler pummelo (used as the female parent of the progenies used for Corsican citron mapping) one self-incompatibility allele at the pollen-specific S-locus F-box (SLF) gene.

The role of structural variants in reproductive isolation between species is an ongoing discussion (Zhang et al., 2021; Berdan et al., 2024), but several biological models highlight the strong negative impact of large structural heterozygosity in male and female fertility (Noor et al., 2001; Baurens et al., 2019). Therefore, the large sexual compatibility between species of the *Citrus* genus whose reticulation occurred 6-8 Ma ago (Wu et al., 2018), as well as the fertility of their interspecific hybrids, can be explained by a highly conserved genomic structure. Large structural variations affecting male and female fertility have, however, been reported in some specific varieties (Ollitrault et al., 2008). It is, for example, the case in the reciprocal translocation in sweet orange cv Valencia, as evidenced by cytogenetic (Iwamasa, 1970) and genome sequencing (Wu et al., 2022). This structural heterozygosity does not result from a structural differentiation between constitutive ancestral species (*C. maxima* and *C. reticulata*) but is due to a mitotic mutational event on a standard sweet orange.

### 4.3 The anchoring of individual and consensus genetic maps on chromosome-scale genome assemblies reveals some discrepancies

The evolution of NGS and particularly of long reads sequencing greatly improved the quality of *de novo* whole genome assembly, and recent publications propose citrus haplotype chromosome-scale assemblies from Nanopore or PacBio HiFi sequencing sometimes coupled with Hi-C sequencing data (Guardo et al., 2021; Wu et al., 2022; Bao et al., 2023; Nakandala et al., 2023, 2024). Our consensus genetic map which is highly syntenic with all individual genetic maps was anchored on 15 chromosome-scale assemblies to identify discrepancies between the consensus map and physical assemblies and therefore between assemblies.

The chromosome numbering and orientation of our consensus genetic map are the same as the first published high density citrus genetic map (Ollitrault et al., 2012) that was adopted for the *C. clementina* v1.0 chromosome assembly, published by the international citrus genome consortium (Wu et al., 2014). The anchoring of the different pseudochromosome assemblies with our individual and consensus maps clarified the relative numbering and orientation of the different genome assemblies (**Supplementary Table 4**) highlighting two mains ways of numbering, deriving from the first two pseudochromosome assemblies (**Supplementary Table 4**): the *C. sinensis* v1.0 (Xu et al., 2013) and the *C. clementina* v1.0 one (Wu et al., 2014). The chromosome orientations appear to be more diverse. This inconsistency in chromosome numbering and orientation may result in confusion regarding the location of useful genes and the integration of QTL information based on different reference genomes or genetic maps.

We analyzed the synteny and collinearity after numbering and orienting all genomes in the same way as in the consensus genetic map. For the same horticultural group, the most recent assembly was the most congruent with the consensus genetic map. This is the case when, for example, comparing the first sweet orange chromosome-level assembly (Xu et al., 2013), the sweet orange V3 assembly (Wang et al., 2021) and the more recent haplotype-resolved assembly of Valencia sweet orange (Wu et al., 2022). Numerous discrepancies were found for synteny with the first chromosome-scale genome assembly of *C. maxima* (Wang et al., 2017), whereas synteny and collinearity were high for the second *C. maxima* assembly (Lu et al., 2022), except for one inverted region of chr9 (consensus map numbering). Similarly, a strong increase in synteny and collinearity was observed between the first haplotype-resolved lemon genomes (Guardo et al., 2021) and the most recent one (Bao et al., 2023). The first lemon haplotype assemblies displayed numerous discrepancies shared with the first *C. maxima* one. It may be explained by the use of this *C. maxima* assembly to finish the chromosome-scale assembly of lemon haplotypes (Guardo et al., 2021).

Therefore, the most recent assemblies for *C. australis*, *C. hindsii*, *C. maxima, C. trifoliata*, sweet orange and lemon appear to be globally highly syntenic and collinear. The discrepancies observed by the anchoring of some genome assemblies with the genetic consensus map may reveal real, large structural variations compared with most citrus species, as well as misplaced regions during the assembly. For the Valencia sweet orange haplotype-resolved assembly (Wu et al., 2022), our results for the preferentially-mandarin haplotype (SwO-USA-A) are fully concordant with the reciprocal translocation revealed by early cytogenetic studies (Iwamasa and Nito, 1988). Moreover, its localization between chr1 (LG1) and chr9 (LG9) is in agreement with the findings of molecular cytogenetic analysis, which localized the translocation between chr4 (LG1 of consensus genetic map) and chr9 (LG9) of the sweet orange V1 assembly (Song et al., 2023). Structural differences are observed between the two published assemblies for *C. trifoliata*. One is highly syntenic and collinear with the consensus map (Peng et al., 2020), whereas the other suggests a translocation of 5 Mb from chr7 (LG7) to chr5 (LG5). It may be interesting to validate the existence of this structural variability in *C. trifoliata* and analyze its potential phenotypical implication. The *C. australis* collapsed assembly is the only one with four telomeric regions (start of chr4 -LG1-, end of chr7 -LG4-, and start and end of chr8 -LG8) not anchored by the consensus genetic map. These genomic regions of the collapsed assembly do not fit well with the individual haplotype assemblies, as shown by the published dot plots between collapsed and haplotype assemblies (Nakandala et al., 2024). The new methodologies combining PacBio HiFi and Hi-C reads have opened the way for telomere-to-telomere gapless assemblies (Sun et al., 2024) and may explain why this very recent genome assembly displays additional telomeric regions when compared with previous citrus assemblies. It may be interesting to validate these identified regions of the collapsed *C. australis* assembly and to analyze if they contain genes not present in the other citrus species and their possible involvement in the resistance/tolerance to HLB of this species (Ramadugu et al., 2016; Alves et al., 2021). For *C. clementina* v1.0 (Wu et al., 2014), previous genetic mapping studies consistently suggested that some genomic regions were misplaced. This is confirmed by the present work with accurate localization (**Figure 4.3**). The concerned genomic regions are (i) from chr5 (12.78-19.40 Mb) and chr4 (4.81-5.84 MB) genetically mapped at the end of LG7; (ii) genomic regions of chr3 (34.30-35.77 Mb) and 9 (11.14-14.45 Mb) genetically mapped in the middle of LG8; (iii) a genomic region of chr2 (0.74-4.23 Mb) mapping at the beginning of LG4; and (iv) a small region of chr8 (16.30-16.71 Mb) mapping at the beginning of LG6. In addition, a misplaced and inverted region is revealed in chr3 (29.03-34.23 Mb). It may be noteworthy that most of these discrepancies (except for the misplaced inverted centromeric region of chr3) concern genomic areas that were not covered by the reference clementine genetic map (Ollitrault et al., 2012) used for the final assembly in pseudochromosomes. The *C. clementina* v1.0 assembly is still used as a reference for several genetic and genomic studies, and our information about the misplaced region may be important for a better interpretation of results.

### 4.4 Perspective for further genetic analysis and interspecific breeding projects

Consensus genetic maps allow researchers to overcome some of the limitations of individual genetic maps. The integration of multiple populations enhances the explored diversity and improves genome coverage thanks to the complementarity of the different families, as a region that is monomorphic within a population may be polymorphic in another (Abed et al., 2022; Fallah et al., 2022; Qu et al., 2021). In citrus, genomic studies revealed identity by descent for numerous genomic regions between mandarins and sweet oranges (Wu et al., 2018) resulting in full homozygosity of large genomic regions in modern mandarins and tangors (mandarin x sweet orange hybrids), as well as large gaps in the genetic maps of these horticultural groups. Our consensus map partially overcomes this problem with an average gap size of 0.36 cM. However, its anchorage in the genome assembly of *C. australis* reveals some uncovered telomeric regions. The consensus may be improved by the integration of additional genetic maps, by taking advantage of the gene-based marker approach.

The combination of data from multiple families also allows researchers to capture more recombination events and therefore increase the mapping resolution (Pootakham et al., 2015; Linge et al., 2018). It therefore improves the precision of QTL analysis and the search for candidate genes and regulatory elements. Our consensus genetic map with 10,756 loci, including 7,915 gene-based markers, will constitute a very useful framework to integrate the locations of QTLs identified from different segregant progenies or genome-wide association studies in various germplasms of the *Citrus* genus. Indeed, we have shown that the rate of successful anchoring on 14 different genomes covering a wide range of the *Citrus* genus diversity, of the 17,718 SNP probes (**Supplementary Table 7**) associated with the 10,756 markers of the consensus map, varies between 91.57% and 99.18%. We can therefore expect to easily infer the location in the consensus map of any QTL or candidate gene identified in the different chromosome-scale genome assemblies.

Consensus genetic maps have also been used to drive the final steps of chromosome-scale assembly (Muñoz-Amatriaín et al., 2011; Mohd Sanusi et al., 2023) and identify large structural genome variations (Khan et al., 2012). The genome structural conservation evidenced by comparative genetic mapping in the *Citrus* genus and anchorage of our consensus genetic map with 15 genomes covering a large diversity of the *Citrus* genus is a favorable situation for further interspecific sexual breeding as well as for translational genomics. It also justifies the use of a unique reference genome to provide a global view of the phylogenomic structure along the genome of modern citrus, as proposed from WGS resequencing (Wu et al., 2014, 2018) or GBS data (Oueslati et al., 2017; Ahmed et al., 2019). However, it appears essential to select a high-quality genome assembly for an accurate determination of interspecific breaking points in the modern citrus genome; our consensus genetic map constitutes a good template to select such high-quality assemblies.

Individual maps remain essential to analyze the distribution of skewed segregation along the genome. It is particularly important in citrus because apomixis by polyembryony (Wang et al., 2017) is present in several species and horticultural groups. Indeed, apomixis has contributed to the accumulation of recessive hidden mutations in heterozygosity, as demonstrated in sweet orange (Wang et al., 2023). The unfavorable mutations can result in gametophytic selection and non-Mendelian segregation. As we observed for our *C. maxima* x *C. reticulata* parent, the linkage drag can extend the skewed segregation in large genomic areas when counter-selected genes are located in regions with low recombination rates. This can be puzzling when interpreting the genetic determinism of useful traits controlled by a gene located in such a region, if only phenotypic segregation data are considered.

Comparative analysis of the nine individual maps and their mapping to the *C. clementina* v1.0 genome assembly revealed a similar recombination landscape for the nine populations and highlighted several large genomic regions with very low recombination rates. This information will be essential in optimizing sexual breeding strategies because these large genomic regions dramatically increase the risk of linkage drag by reducing the probability of recombination between useful and unfavorable genes.

## 5 Conclusion

The GBS data of 1,216 hybrids from 10 bi-parental families using the *Ape*K I restriction enzyme were powerful in developing nine uni-parental high-density genetic maps encompassing five ancestral species of the *Citrus* genus, two horticultural groups resulting from interspecific admixture and two F1 interspecific hybrids. The predominance of the cutting site of *Ape*K I in gene sequences permitted the development of a consensus map based on common gene-based markers between the various individual maps. It spans 1,005.27 cM and includes 10,756 loci: 7,915 being gene-based markers and 2,841 SNPs located-out gene sequences. The synteny is complete between the consensus map and the individual maps, and their collinearity is very high. The comparative genetic mapping and anchoring of the consensus map on 15 published chromosome-scale genome assemblies highlighted the differences in numbering and orientation of chromosomes within different genome assemblies. Once these parameters were homogenized, the consensus genetic map appeared to be highly syntenic and collinear with the most recent genome assemblies, whereas discrepancies were observed for some older ones. These high synteny and collinearity concern the recent genome assemblies of *C. australis, C. hindsii, C. maxima, C. trifoliata*, *C.* x*. aurantium* var. *sinensis* and *C.* x*. lemon* var. *lemon*. The role of large structural variations during speciation within the *Citrus* genus seems to have been limited. It may explain the high level of sexual compatibility between most *Citrus* species and the frequent good fertility of the F1 interspecific hybrids. The recombination landscape also appears to be largely conserved between ancestral species and F1 interspecific hybrids. Large genomic regions with very low recombination rates have been identified. This information will be very useful to estimate the linkage drag extent and to optimize conventional breeding schemes. Non-Mendelian segregations are frequent in citrus and were observed in specific regions for each parental combination. They may complicate the interpretation of a useful traits’ genetic determinants. Our consensus genetic map constitutes the most saturated genetic framework published in citrus, and its congruence with the most recent citrus genome assemblies validates its quality. It may be easily extended to other species and horticultural groups, taking advantage of the gene-based marker approach. The consensus genetic map is a useful tool to check the accuracy of genome assemblies, identify large structural variation that may have occurred within the horticultural group (such as that of Valencia sweet orange) and provide a focus to study potential relationships with phenotypic variations. It may also be a reference framework to integrate the positions of QTLs and useful genes identified from different segregant progenies or genome-wide association studies in various germplasms of the *Citrus* genus.

## Data Availability

The demultiplexed raw data of Genotyping By Sequencing (GBS) have been deposited at NCBI under BioProject numbers: PRJNA1119914, PRJNA1120630, PRJNA1120237, PRJNA1120896, PRJNA1124254, PRJNA1125292, PRJNA1125912, PRJNA1128137, PRJNA1128404. Detailed data about individual and consensus genetic maps and their anchoring on 15 published genome assemblies (including probe sequence of the consensus genetic map) are provided as **Supplementary Material**.
