# Supplementary material for: Comparative genetic mapping and a consensus interspecific genetic map reveal strong synteny and collinearity within the Citrus genus
Source: Front Plant Sci. 2024 Dec 16;15:1475965. doi: 10.3389/fpls.2024.1475965 (PMC11682908; doi:10.3389/fpls.2024.1475965)
Supplement: Supplementary file 1 [file DataSheet1.pdf]

Ollitrault et al., Comparative genetic mapping and a consensus interspecific genetic map reveals strong synteny and collinearity within the Citrus genus. Frontiers in Plant Science.

Supplementary figure 1: Marey map of the genetic maps relative to *C. clementina* V1.0 genome assembly

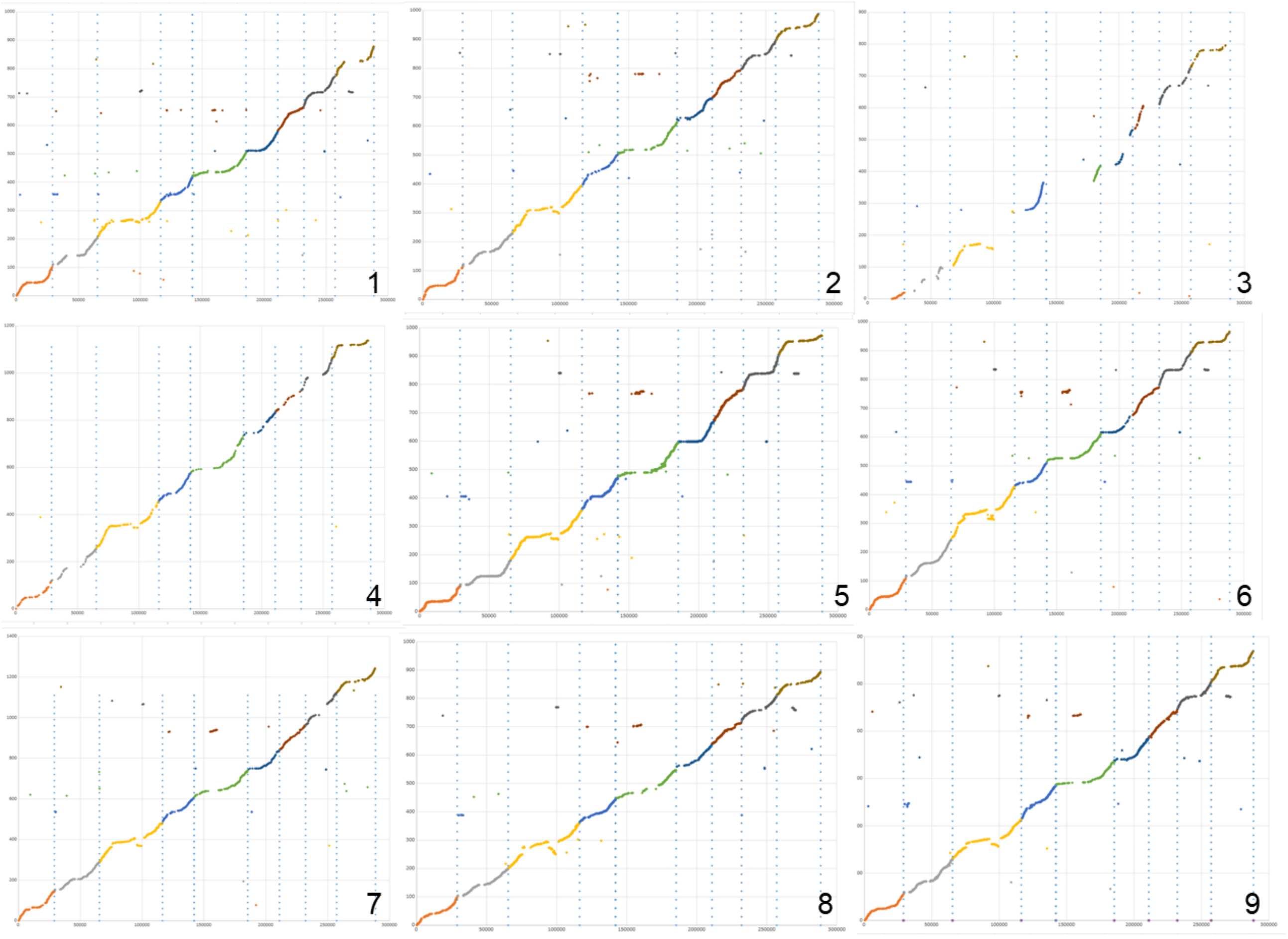

Supplementary Figure 1a: Marey Map of the nine genetic maps relative to C. clementina V1.0 genome assembly

X axis physical position on C. clementina V1.0 reference genome ; Y axis: genetic position (cM)

1: *C. maxima*, 2: *C. reticulata*, 3: *C. medica*, 4: *C. x aurantium var clementina*, 5: *C. x limon*, 6: C. maxima x C. reticulata, 7: *C. trifoliata*, 8: *C. glauca*, 9: *C. australis x C. inodora*

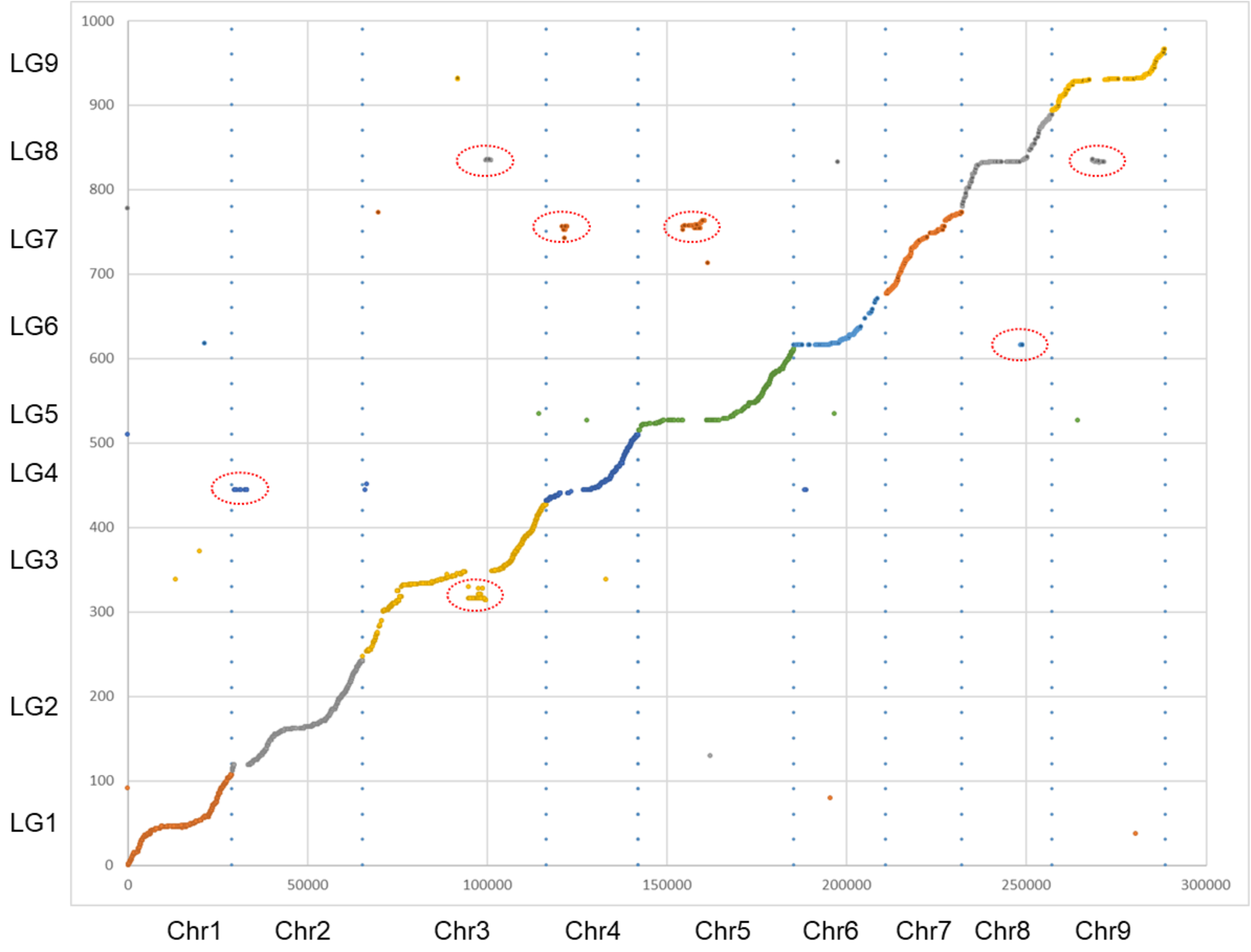

Supplementary figure 1b: Marey Map of the *C. maxima x C. reticulata* genetic map, relative to *C. clementina* V1.0 genome assembly

X axis physical position on *C. clementina* V1.0 reference genome ; Y axis: genetic position (cM)

Red ellipse discrepancies with the *C. clementina* V1.0 assembly shared with others genetic maps
