# Supplementary material for: Comparative genetic mapping and a consensus interspecific genetic map reveal strong synteny and collinearity within the Citrus genus
Source: Front Plant Sci. 2024 Dec 16;15:1475965. doi: 10.3389/fpls.2024.1475965 (PMC11682908; doi:10.3389/fpls.2024.1475965)
Supplement: Supplementary file 2 [file DataSheet2.pdf]

Ollitrault et al., Comparative genetic mapping and a consensus interspecific genetic map reveals strong synteny and collinearity within the Citrus genus. Frontiers in Plant Science.

Supplementary Figure 2: Gene density, marker density, recombination landscape, and skewed segregation

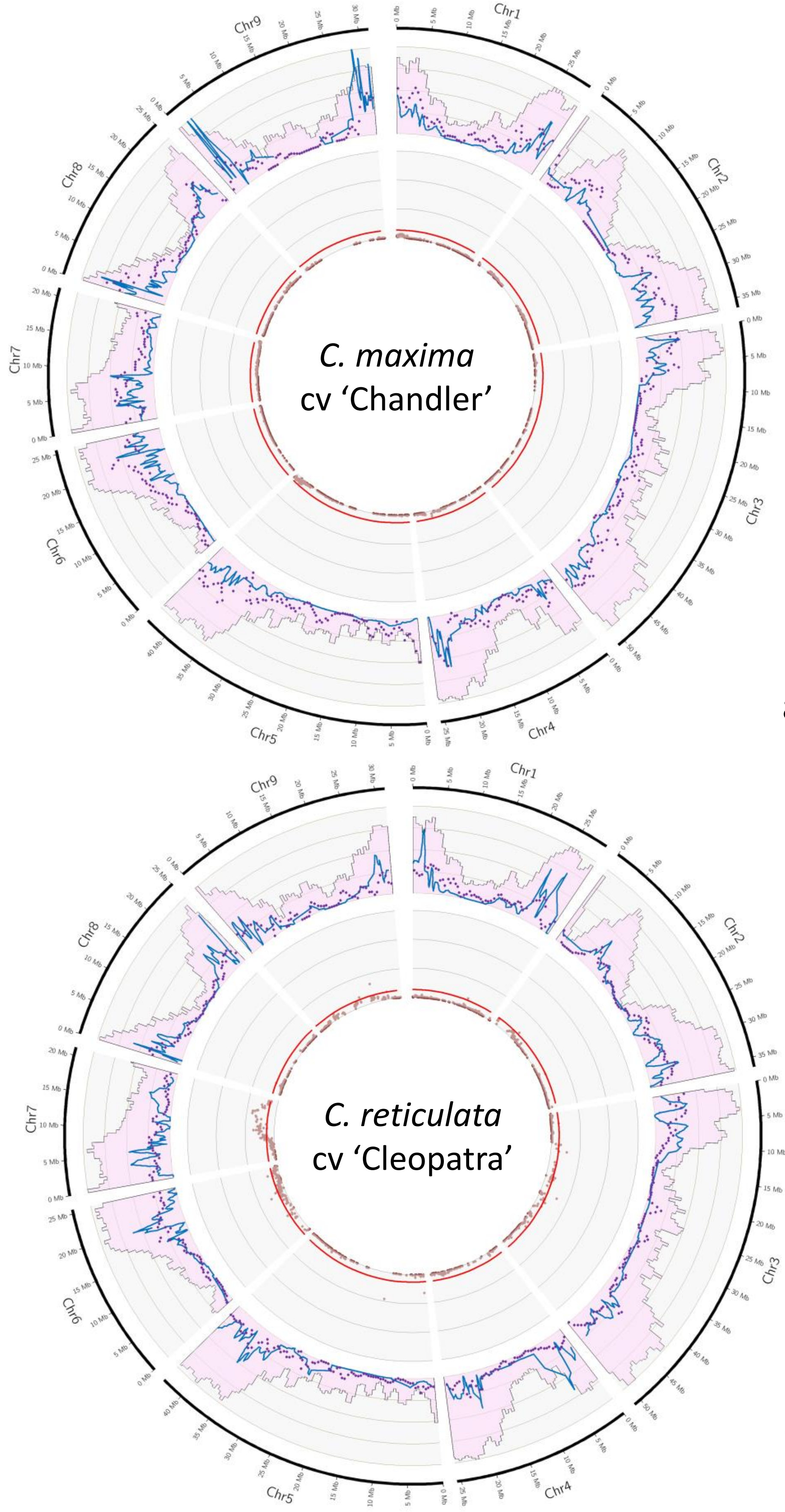

a

b

External Outer ring: Pink histogram: gene density (scale 0-50%), blue line local recombination (scale 0-30 cM/Mb; purple dot: number of markers (scale: 0-5/100kb); inner ring: red line Threshold for Qvalue (0.05) significance; brown dot: Qvalue for Mendelian segregation (scale: 0-15)

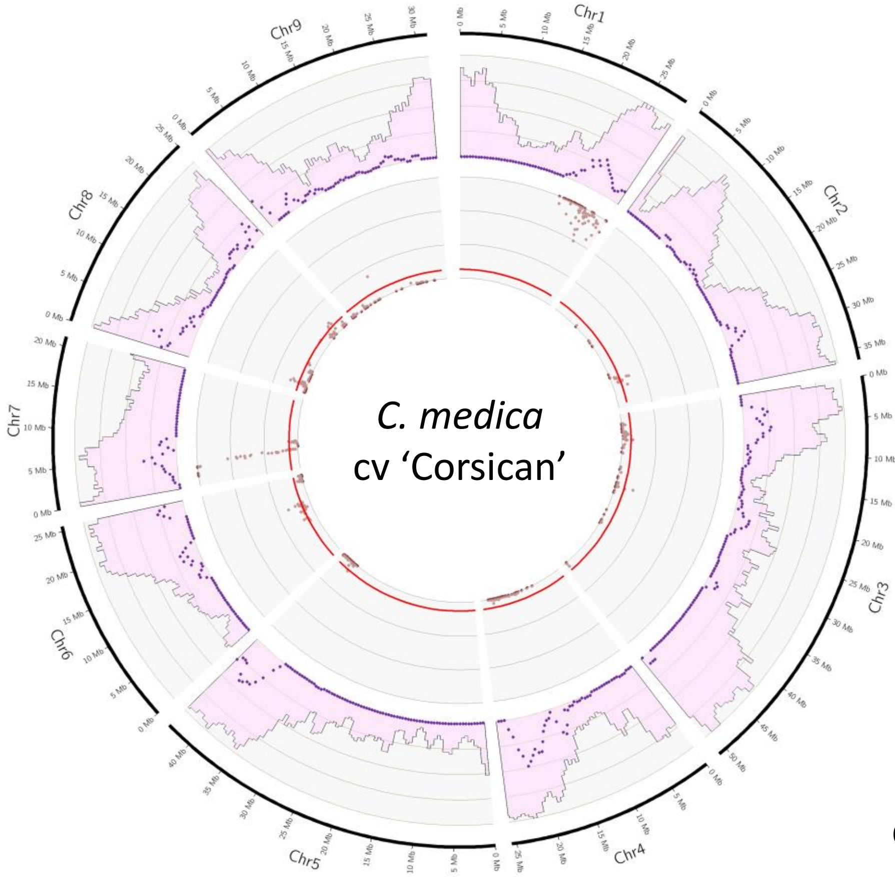

c

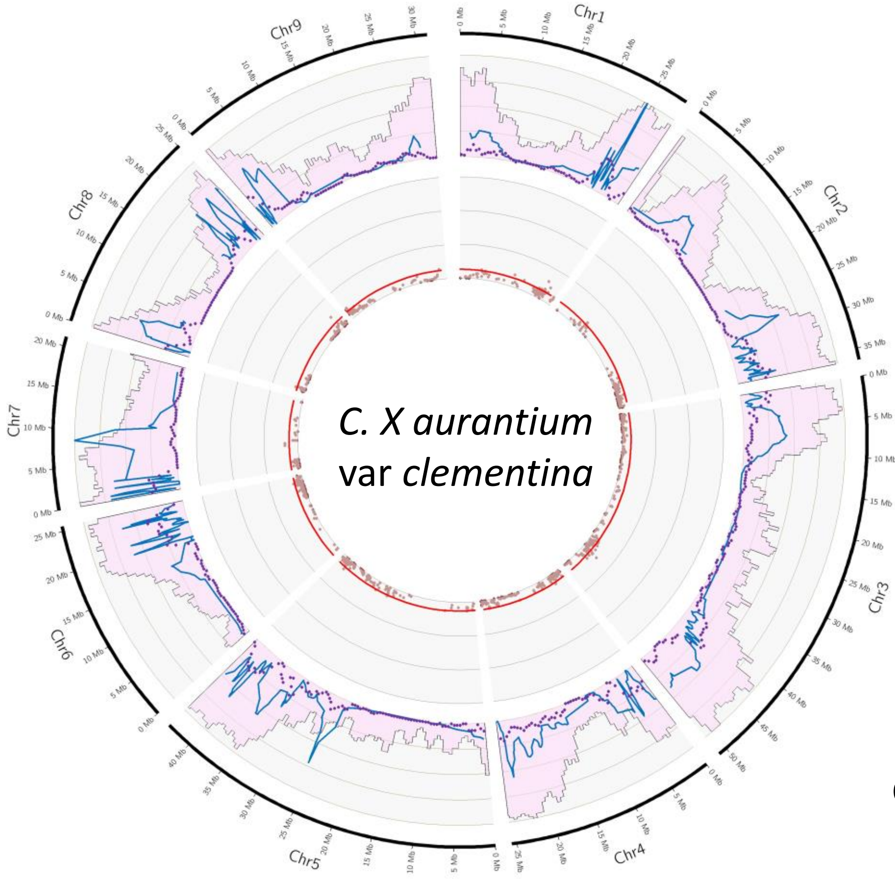

d

External Outer ring: Pink histogram: gene density (scale 0-50%), blue line local recombination (scale 0-30 cM/Mb; purple dot: number of markers (scale: 0-5/100kb); inner ring: red line Threshold for Qvalue (0.05) significance; brown dot: Qvalue for Mendelian segregation (scale: 0-15)

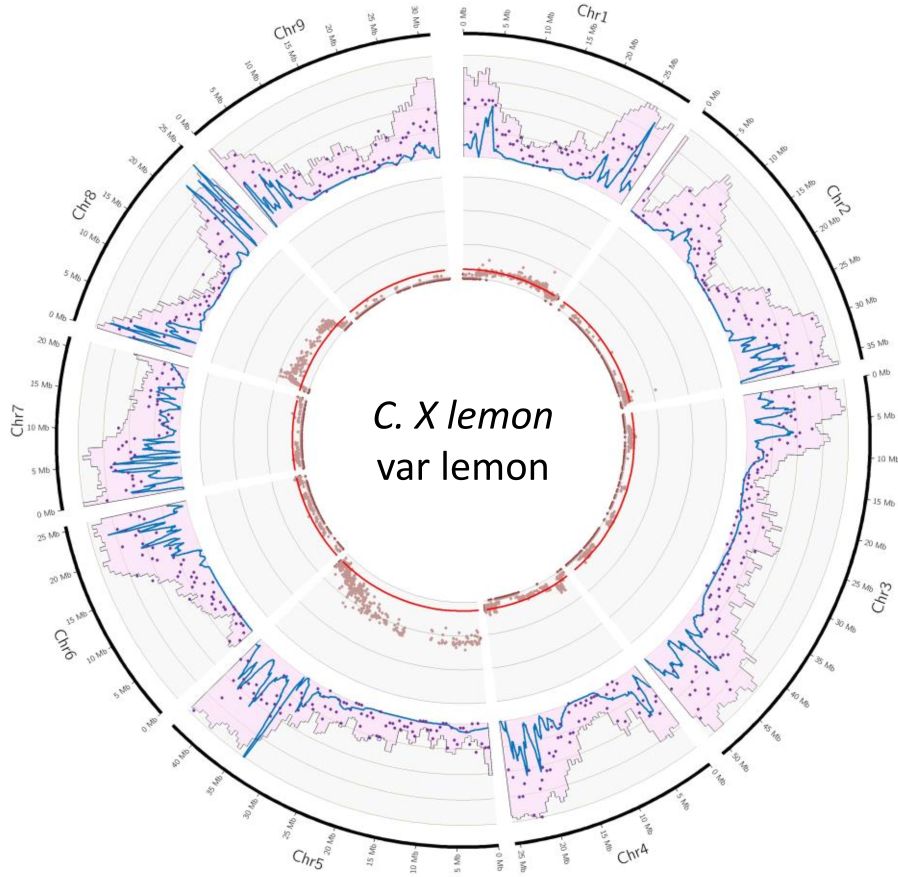

e

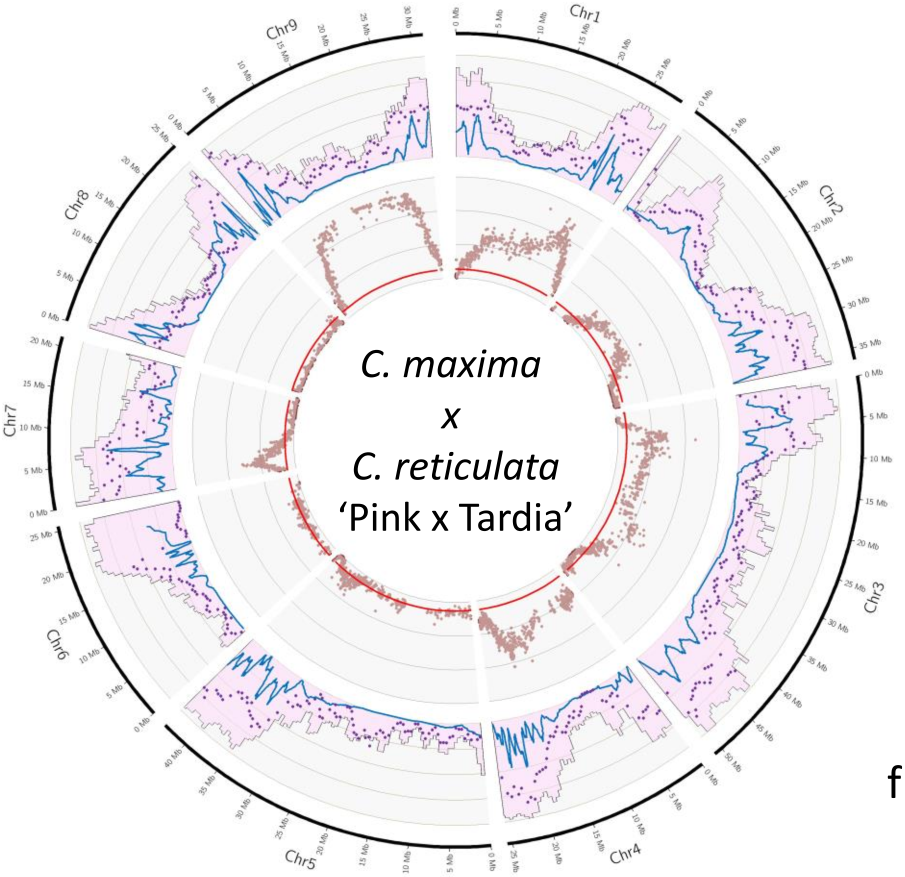

f

External Outer ring: Pink histogram: gene density (scale 0-50%), blue line local recombination (scale 0-30 cM/Mb; purple dot: number of markers (scale: 0-5/100kb); inner ring: red line Threshold for Qvalue (0.05) significance; brown dot: Qvalue for Mendelian segregation (scale: 0-15)

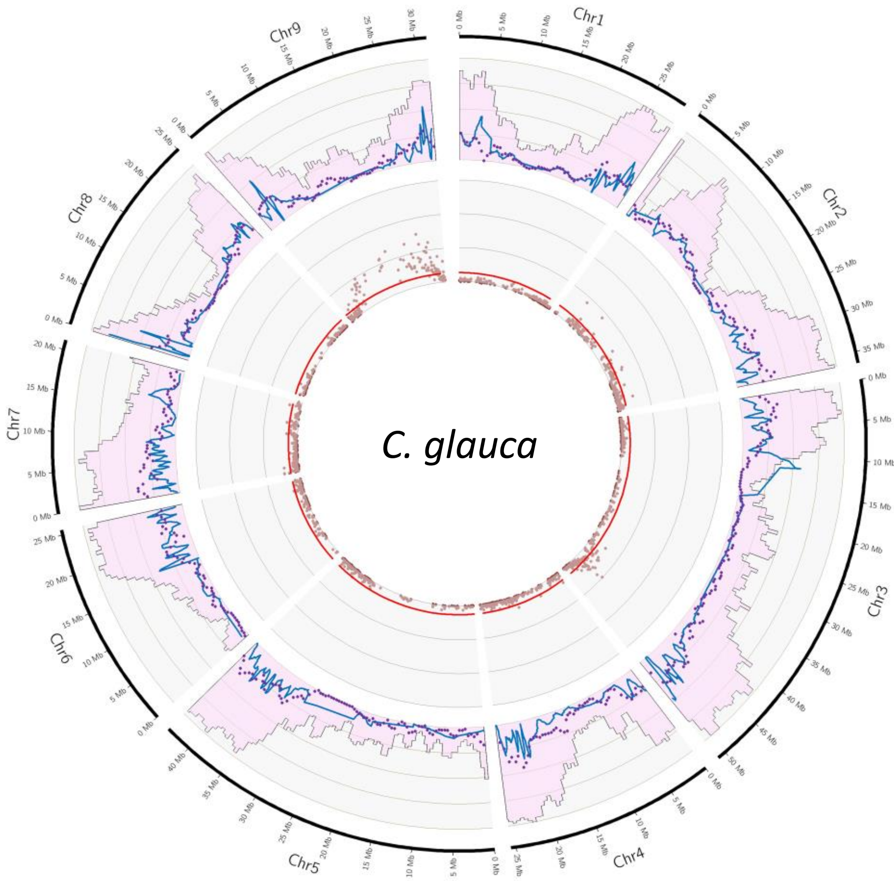

g

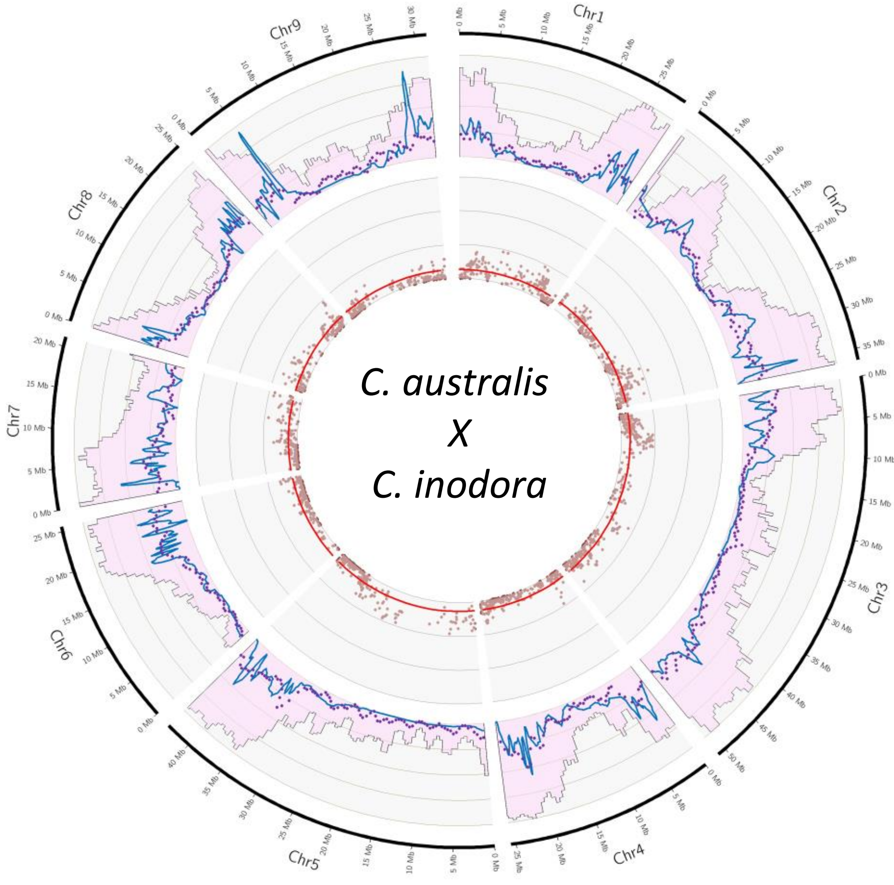

h

External Outer ring: Pink histogram: gene density (scale 0-50%), blue line local recombination (scale 0-30 cM/Mb; purple dot: number of markers (scale: 0-5/100kb); inner ring: red line Threshold for Qvalue (0.05) significance; brown dot: Qvalue for Mendelian segregation (scale: 0-15)

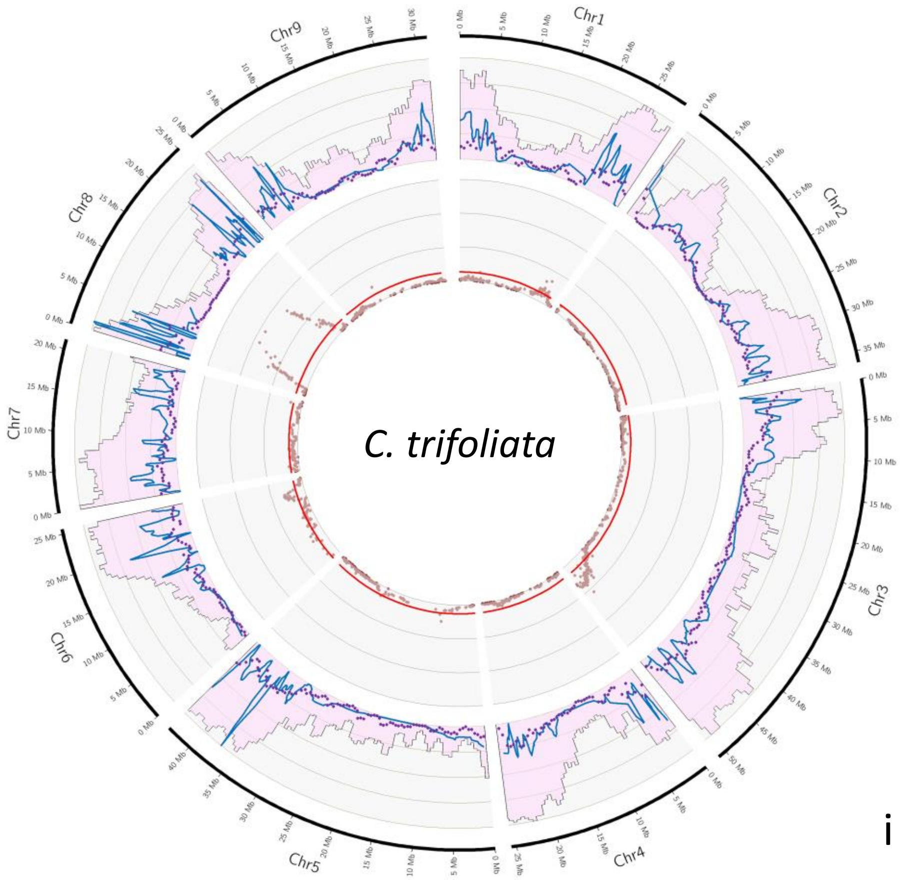

i

External Outer ring: Pink histogram: gene density (scale 0-50%), blue line local recombination (scale 0-30 cM/Mb; purple dot: number of markers (scale: 0-5/100kb); inner ring: red line Threshold for Qvalue (0.05) significance; brown dot: Qvalue for Mendelian segregation (scale: 0-15)
