# Supplementary material for: Comparative genetic mapping and a consensus interspecific genetic map reveal strong synteny and collinearity within the Citrus genus
Source: Front Plant Sci. 2024 Dec 16;15:1475965. doi: 10.3389/fpls.2024.1475965 (PMC11682908; doi:10.3389/fpls.2024.1475965)
Supplement: Supplementary file 3 [file DataSheet3.pdf]

Ollitrault et al., Comparative genetic mapping and a consensus interspecific genetic map reveals strong synteny and collinearity within the Citrus genus. Frontiers in Plant Science.

**Supplementary Figure 3:** Link between genetic maps and related genome assemblies.

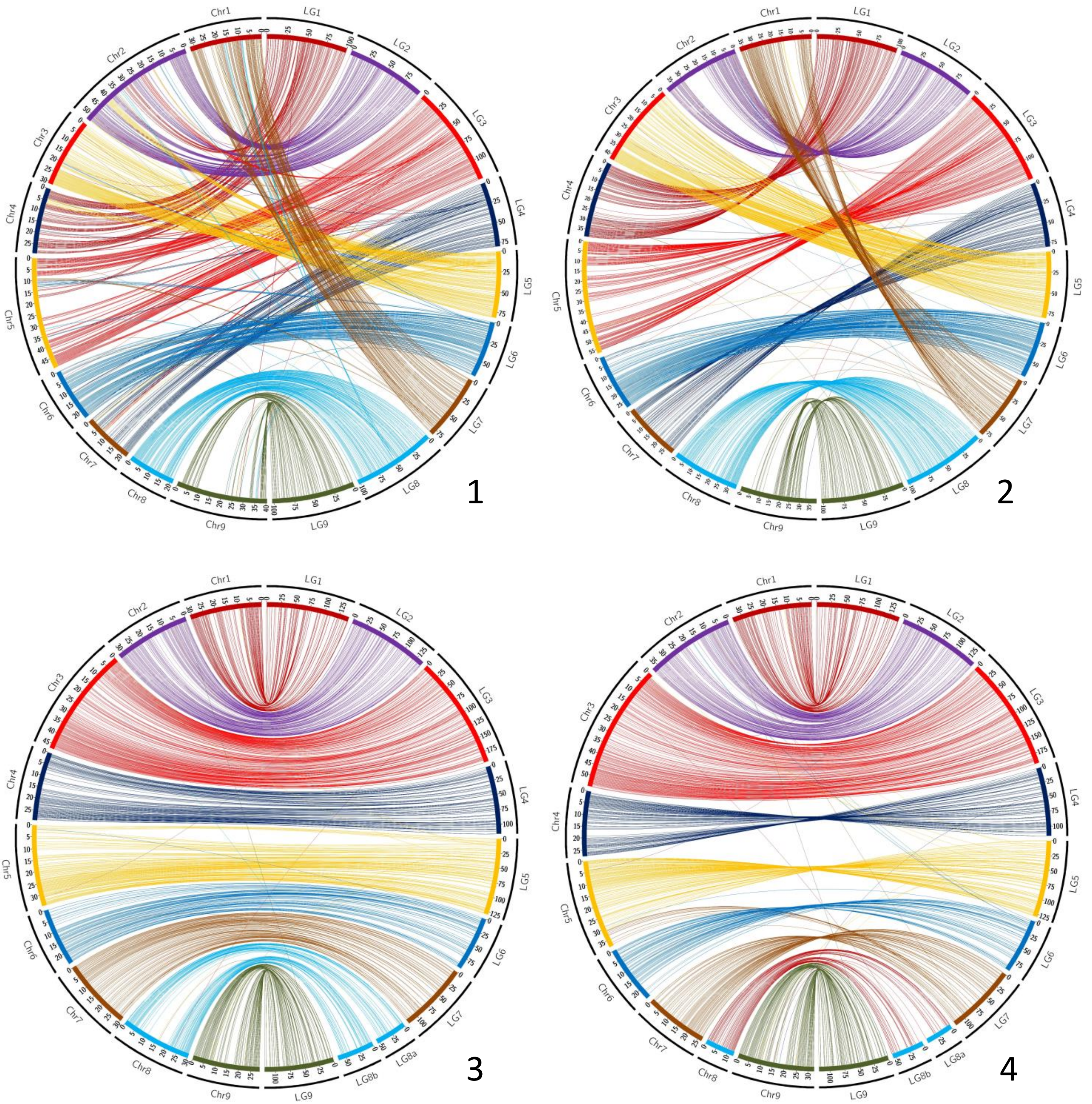


Supplementary Figure 3a: Link between genetic maps and related genome assemblies.
1: C. maxima map / Pum-China-V1; 2: C. maxima map / Pum-China-V2
3: C. trifoliata map / TOr-USA; 4: C. trifoliata map / Tor-China

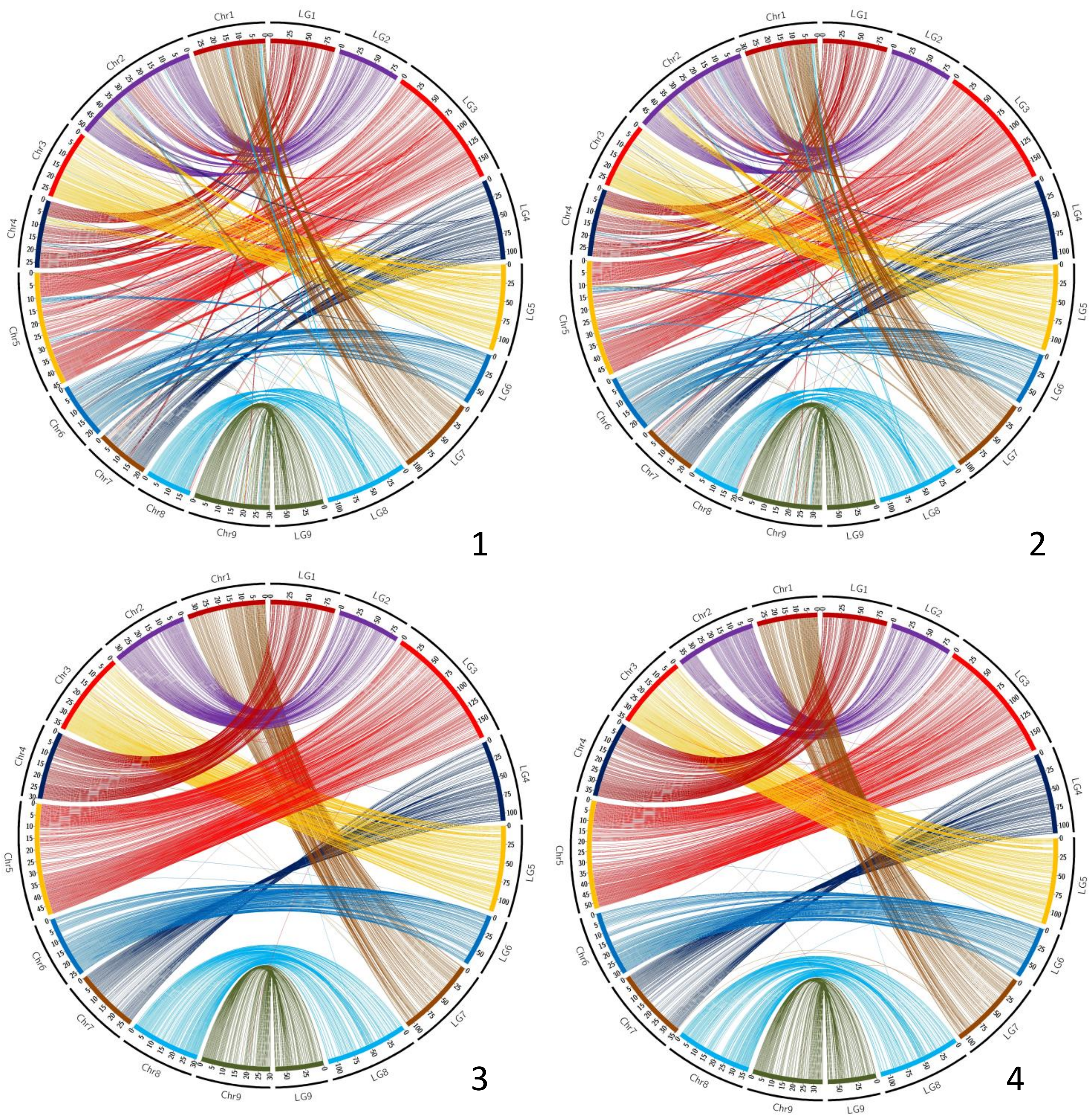


Supplementary Figure 3b: Link between genetic maps and related genome assemblies.
1: C. x limon map / Lemon-It-Prim;  2: : C. x limon map / Lemon-It-Alt
3: C. x limon map / Lemon-China-HapA; 4: C. x limon map / Lemon-China-HapB

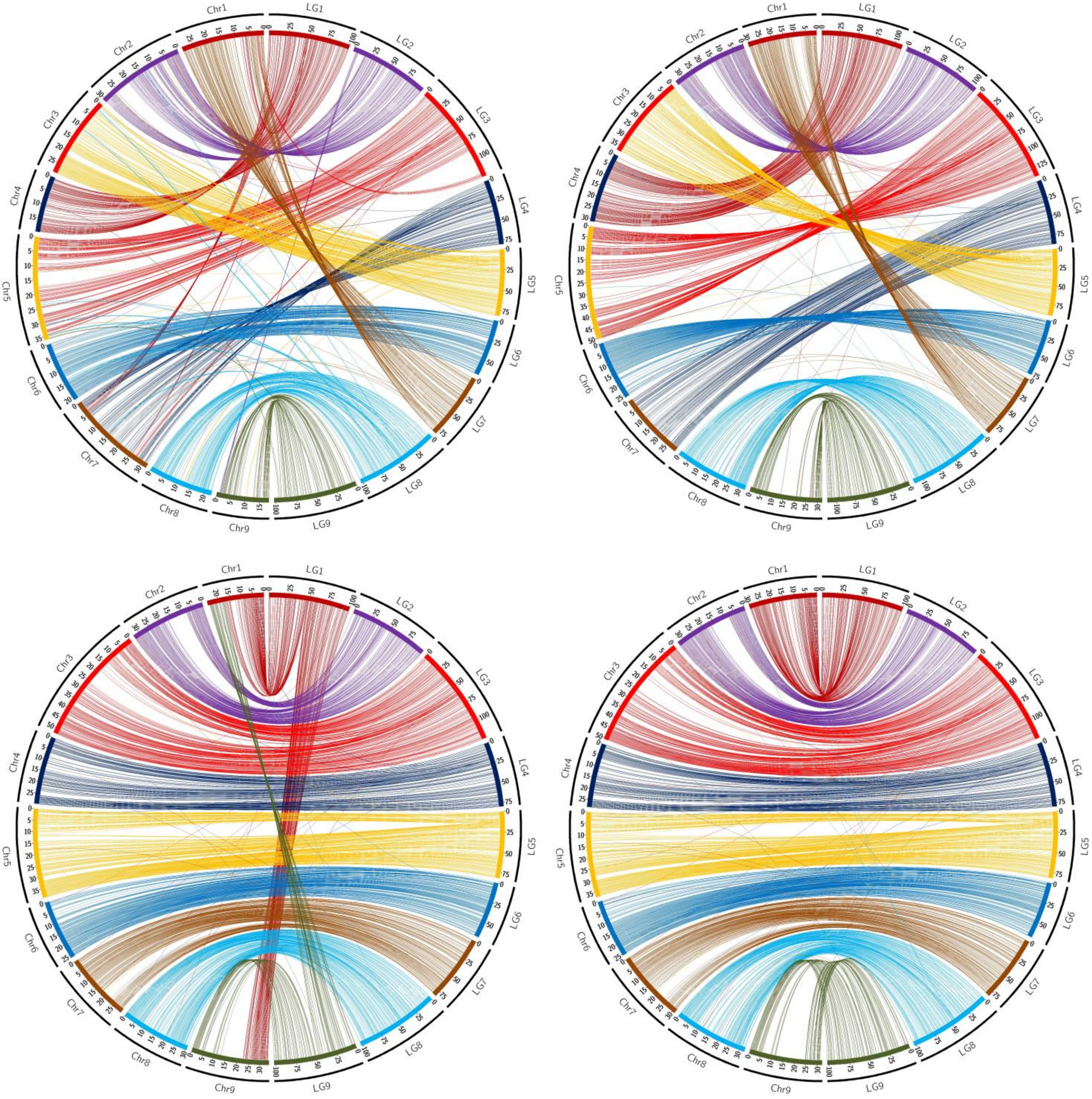


Supplementary Figure 3c: Link between genetic maps and related genome assemblies.
1: C. maxima map / SwO-China-V1;  2: : C. maxima map / SwO-China-V3
3: C. maxima map / SwO-USA-A; 4: C. maxima map / SwO-USA-B

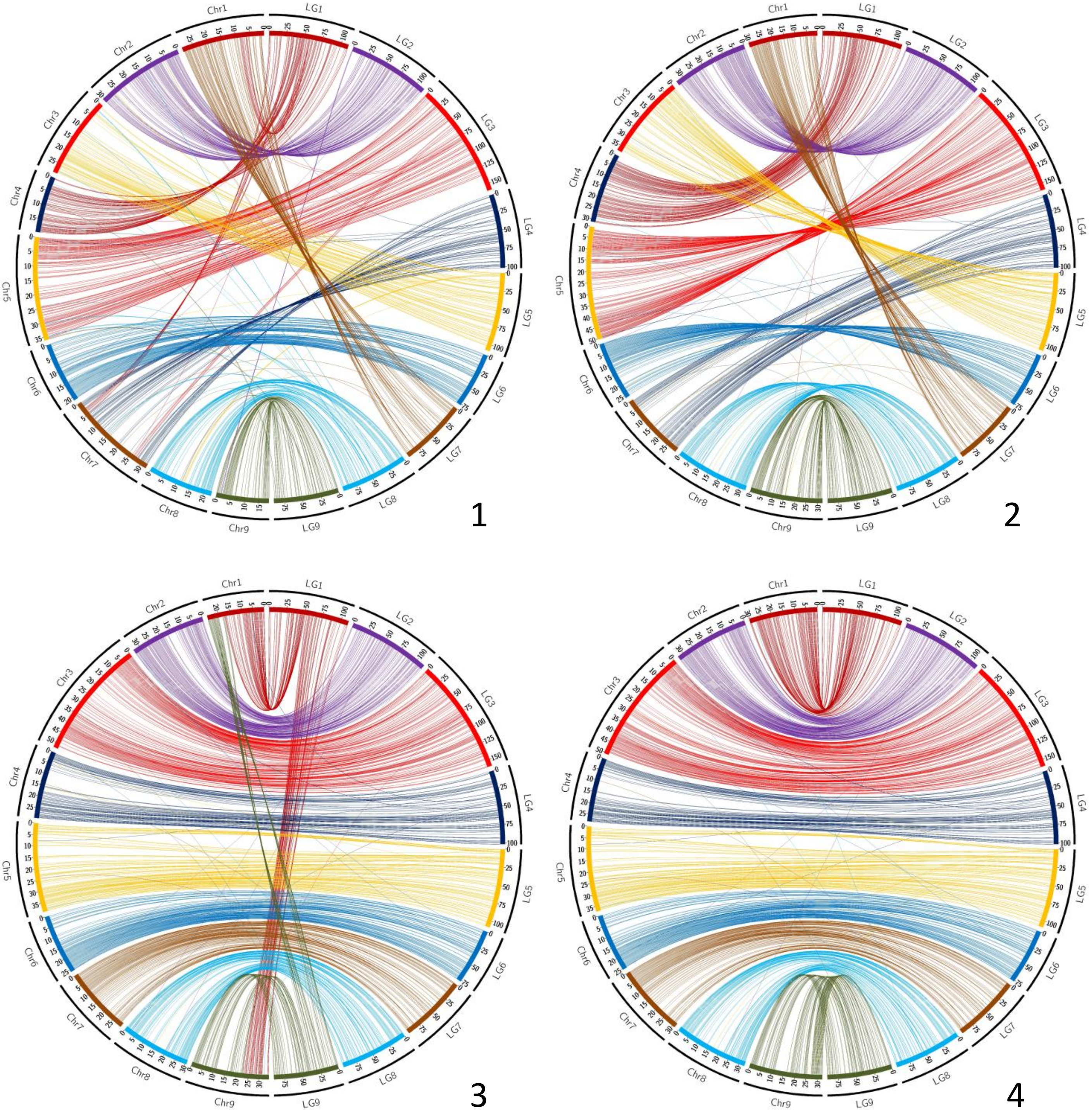


Supplementary Figure 3d: Link beetween genetic maps and related genome assemblies.
1: C. reticulata map / SwO-China-V1;  2: : C. reticulata map / SwO-China-V3
3: C. reticulata map/ SwO-USA-A; 4: C. reticulata map/ SwO-USA-B

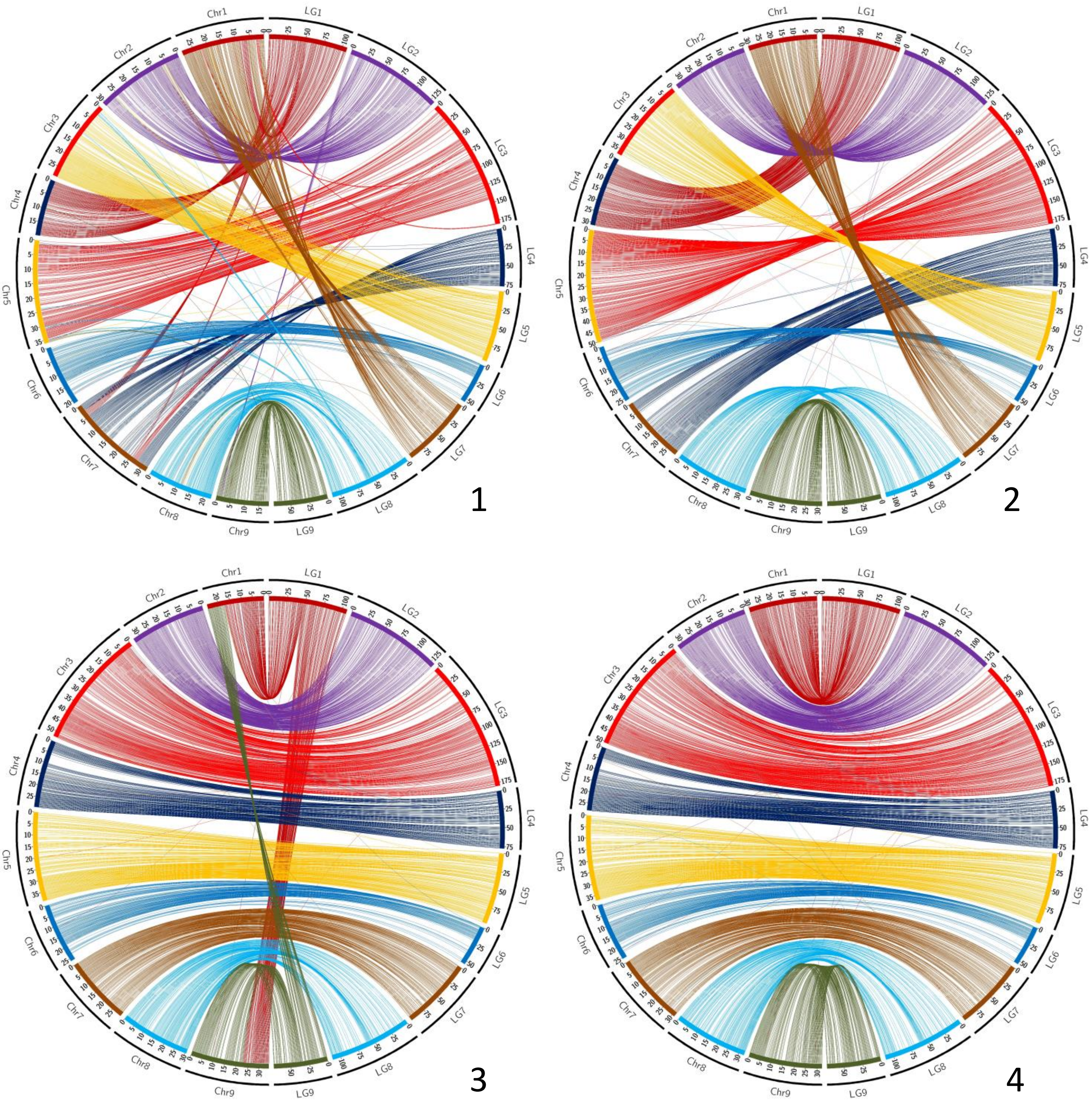


Supplementary Figure 3e: Link between genetic maps and related genome assemblies.
1: C. maxima x C. reticulata / SwO-China-V1; 2: C. maxima x C. reticulata / SwO-China-V3
3: C. maxima x C. reticulata / SwO-USA-A; 4: C. maxima x C. reticulata / SwO-USA-B

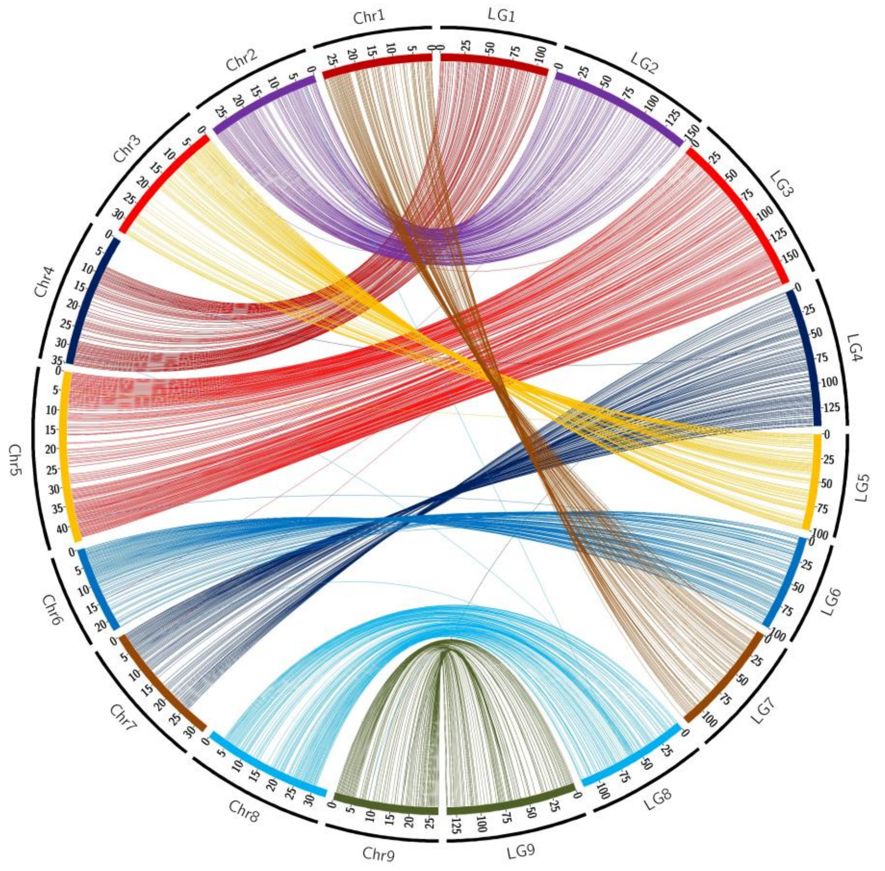


Supplementary Figure 3f: Link between genetic maps and related genome assemblies.
C. australis x C. inodora genetic map / C. australis assembly
