# Supplementary material for: Comparative genetic mapping and a consensus interspecific genetic map reveal strong synteny and collinearity within the Citrus genus
Source: Front Plant Sci. 2024 Dec 16;15:1475965. doi: 10.3389/fpls.2024.1475965 (PMC11682908; doi:10.3389/fpls.2024.1475965)
Supplement: Supplementary file 4 [file DataSheet4.pdf]

Ollitrault et al., Comparative genetic mapping and a consensus interspecific genetic map reveals strong synteny and collinearity within the Citrus genus. Frontiers in Plant Science.

Supplementary Figure 4: Comparison of Pink x Tardia genetic maps and the eight other maps

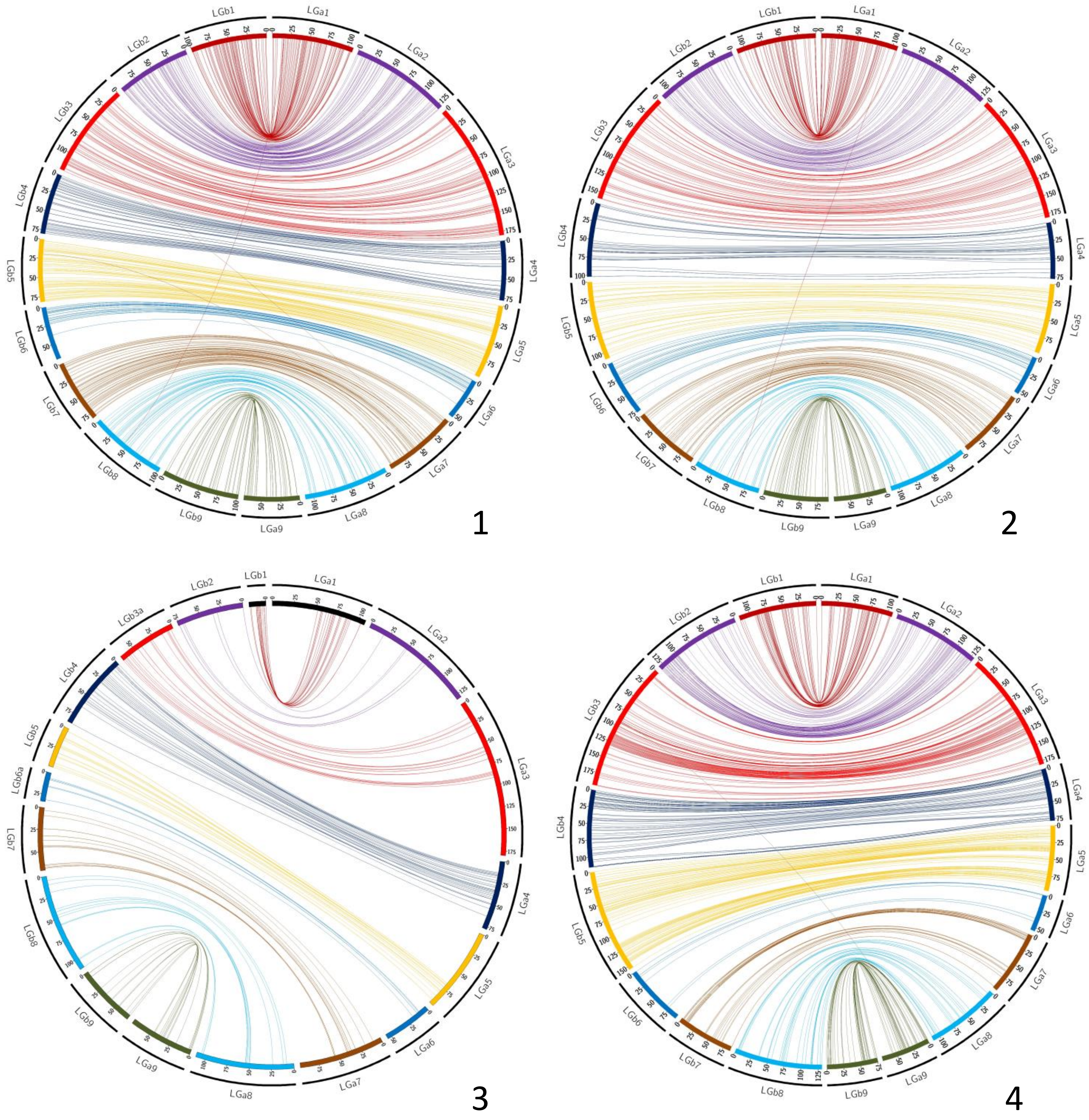

1: *C. maxima*; 2: *C. reticulata*; 3: *C. medica*; 4: *C. x aurantium var clementina*

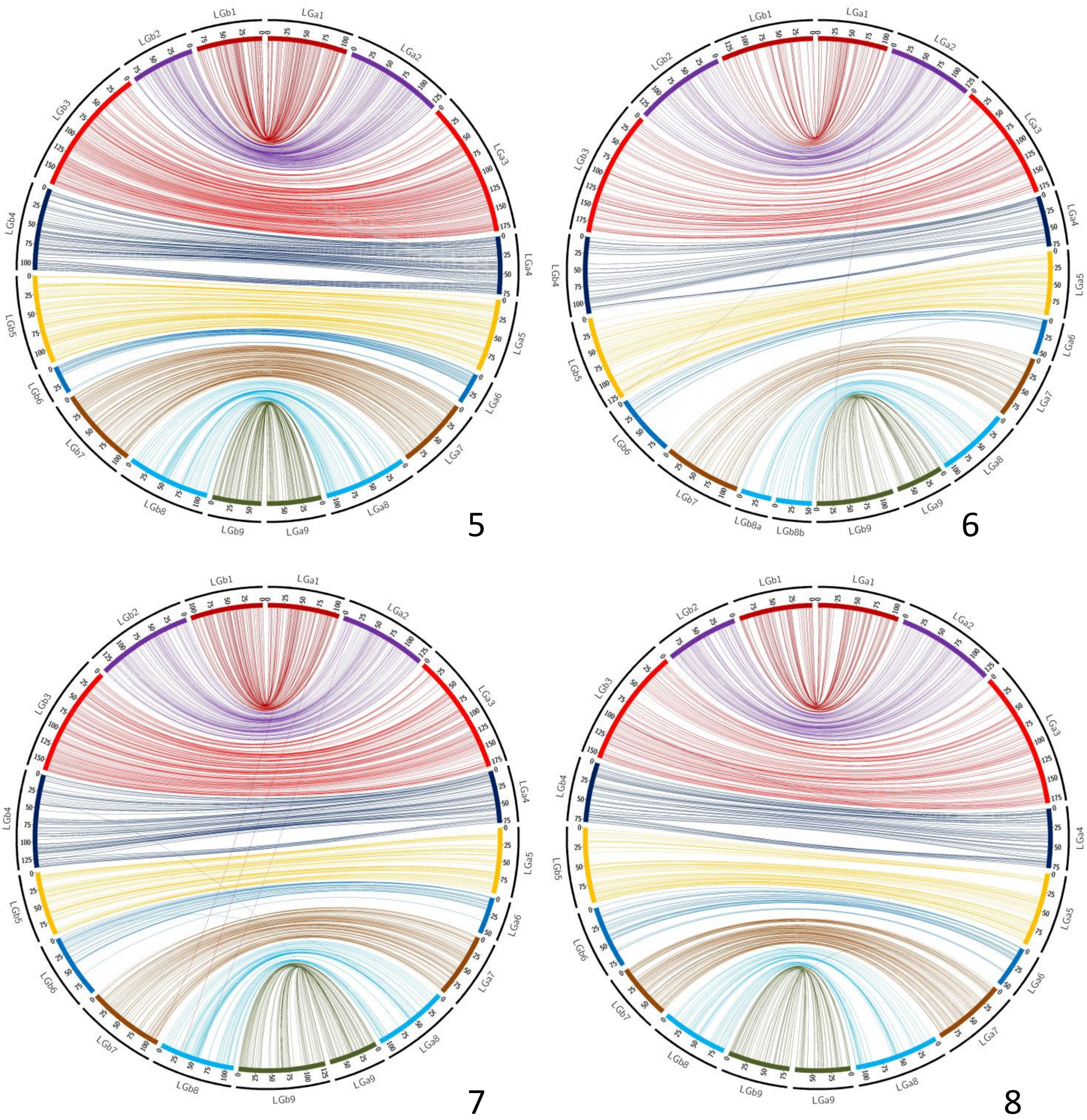

5: *C. x limon*; 6: *C. trifoliata*; 7: *C. australis x C. inodora*; 8: *C. glauca*
